# Expression and Purification of Cp3GT: Structural Analysis and Modeling of a Key Plant Flavonol-3-O Glucosyltransferase from *Citrus paradisi*

**DOI:** 10.3390/biotech13010004

**Published:** 2024-02-07

**Authors:** Aaron S. Birchfield, Cecilia A. McIntosh

**Affiliations:** Department of Biological Sciences, East Tennessee State University, P.O. Box 70703, Johnson City, TN 37614, USA; birchfieldas@etsu.edu

**Keywords:** glycosyltransferases (GTs), flavonols, recombinant protein production, *Pichia pastoris*, protein purification, structural analysis, in silico modeling, enzyme–substrate interactions, synthetic biology

## Abstract

Glycosyltransferases (GTs) are pivotal enzymes in the biosynthesis of various biological molecules. This study focuses on the scale-up, expression, and purification of a plant flavonol-specific 3-O glucosyltransferase (Cp3GT), a key enzyme from *Citrus paradisi*, for structural analysis and modeling. The challenges associated with recombinant protein production in *Pichia pastoris*, such as proteolytic degradation, were addressed through the optimization of culture conditions and purification processes. The purification strategy employed affinity, anion exchange, and size exclusion chromatography, leading to greater than 95% homogeneity for Cp3GT. In silico modeling, using D-I-TASSER and COFACTOR integrated with the AlphaFold2 pipeline, provided insights into the structural dynamics of Cp3GT and its ligand binding sites, offering predictions for enzyme–substrate interactions. These models were compared to experimentally derived structures, enhancing understanding of the enzyme’s functional mechanisms. The findings present a comprehensive approach to produce a highly purified Cp3GT which is suitable for crystallographic studies and to shed light on the structural basis of flavonol specificity in plant GTs. The significant implications of these results for synthetic biology and enzyme engineering in pharmaceutical applications are also considered.

## 1. Introduction

Glycosyltransferases (GTs) are enzymes that catalyze the transfer of sugar moieties from activated donor molecules to specific acceptor molecules, playing vital roles in the biosynthesis of carbohydrates, lipids, proteins, and secondary metabolites. They are ubiquitous across the plant and animal kingdoms, and their functions are diverse and fundamental to various biological processes. In plants, GTs are particularly essential for the glucosylation of secondary metabolites, including flavonoids. Flavonoid glycosides serve crucial roles for plants, offering protection against herbivores, pathogens, and environmental stressors, as well as providing protection from ultra-violet light and imparting unique taste and color profiles to edibles [1,2,3,4,5,6,7,8,9]. Furthermore, these glycosides have garnered significant attention in the human health domain due to their roles in modulating inflammatory pathways, neutralizing reactive oxygen species, and inhibiting tumor cell proliferation [10,11,12,13,14,15].

The rapidly advancing field of synthetic biology has cast a spotlight on the instrumental role of GTs, particularly in the realms of enzyme engineering and the tailored synthesis of complex molecules. Many GTs exhibit exceptional regio-, stereo-, and substrate specificities, making them invaluable tools for the forming of specific glycosidic bonds [16,17,18,19]. Through rational mutagenesis and directed evolution strategies, GTs have been engineered to facilitate the efficient synthesis of a diverse array of complex carbohydrates, glycoproteins, antibiotics, and herbal extracts with medicinal value [20,21,22,23,24,25,26]. The potential implications of these bioengineered molecules are profound, particularly in the pharmaceutical industry, where they play pivotal roles in drug discovery and therapeutic developments [27,28,29].

A flavonol-specific 3-O glucosyltransferase (Cp3GT, Genbank Accession ID–ACS15351.1) was identified in *Citrus paradisi* and shown through biochemical characterization to preferentially glucosylate the flavonols quercetin, kaempferol, myricetin, and fisetin at the 3-OH position [30,31]. This enzyme exhibits a unique specificity among glycosyltransferases, focusing exclusively on the 3-OH position of flavonols, a feature not reported in other GTs. Mutational and in silico analyses suggested essential residues that play crucial roles in substrate docking and 3-O glucosylation [32,33]. Sequence alignments further highlighted that Cp3GT shares a 56.7% sequence identity with a uridine diphosphate glycosyltransferase (UGT) from *Vitis vinifera* (VvGT1, PDB ID–2C1Z) and a 43% sequence identity with a UGT from *Clitoria ternatea* (UGT78K6, PDB ID–4REN). While VvGT1 can glucosylate both flavonols and anthocyanidins, UGT78K6 is specialized for the glucosylation of anthocyanidins alone. In contrast, Cp3GT exclusively catalyzes the glucosylation of flavonols, underscoring its potential as a model for understanding substrate specificity in GTs [34,35,36].

A notable point of convergence among these glucosyltransferases is the conservation of the N-terminal histidine residue—specified as His20 in VvGT1, His22 in Cp3GT, and His17 in UGT78K6—which is critical for the 3-O glucosylation process [33,34,36]. The presence of this conserved catalytic residue in enzymes with diverse substrate specificities suggests that other structural elements or residues must play determinant roles in substrate recognition and specificity. To decipher the mechanistic underpinnings of these specificities, an in-depth structural and functional analysis of the binding pocket and the residues therein is warranted. Understanding the spatial configuration and properties of these residues can provide insights into substrate orientation and docking dynamics.

The most recent Cp3GT in silico analysis, conducted in 2018, relied on EasyModeller for structure prediction and AutoDock Vina for docking [33]. Since then, significant advances in structure prediction methodologies have been made. Notably, the emergence of deep learning and machine learning-driven artificial intelligence tools such as I-TASSER and AlphaFold have revolutionized the field [37,38]. Unlike conventional modeling tools such as EasyModeller, I-TASSER and AlphaFold employ advanced deep learning algorithms that provide a more dynamic understanding of protein folding [37,38]. These algorithms can accurately predict intricate local and global structural patterns in proteins and outperform traditional techniques that primarily depend on template-based modeling.

Given the evolution of these newer, more advanced tools, it would be beneficial to revisit Cp3GT structural models and docking simulations. By comparing these with earlier models, analogous GTs with resolved crystal structures, and correlating them with biochemical insights from GT activity and mutational assays, a consolidated understanding of Cp3GT’s structure can be achieved, potentially unearthing novel insights.

The use of the *Pichia pastoris* system for producing recombinant proteins is popular due to its yield and scalability. However, its endogenous proteases, including serine, aspartic, and metalloproteases, pose challenges by potentially degrading expressed proteins and altering their structural integrity [39,40,41,42]. Such degradation can affect a protein’s stability and activity and its behavior during purification, leading to variability in enzymatic studies [43,44,45]. To counteract protease action, strains with reduced protease activity or those that are genetically modified to eliminate specific protease genes, such as PEP4, PRB1, and YPSs, are utilized [46]. Moreover, the adjustment of culture conditions and the integration of protease inhibitors such as phenylmethylsulfonyl fluoride (PMSF) have shown efficacy in minimizing protein degradation [30,31,43,47,48].

This research focused on the feasibility of scaling up the production of recombinant Cp3GT using the methanol-inducible *Pichia pastoris* system and aimed to produce sufficient quantities of the protein for comprehensive analysis. It was demonstrated that Cp3GT could be effectively purified to a homogeneity of ≥95% using a combination of standard chromatographic techniques, including affinity, anion exchange, and size-exclusion chromatography.

Additionally, the study investigated the challenge of recombinant Cp3GT degradation by endogenous *Pichia pastoris* proteases during the purification process. These findings indicated the potential for proteolytic degradation, which might lead to a truncated form of the protein, potentially affecting GT catalysis and compromising the integrity of the C-terminal c-myc/6x His tags critical for purification and identification.

Furthermore, a detailed in silico structural analysis of Cp3GT was conducted as part of the research approach. Utilizing advanced modeling and docking techniques, this study aimed to identify key residues essential for flavonol binding and its subsequent 3-O glucosylation. This approach was designed to deepen the understanding of the molecular mechanisms underlying Cp3GT function.

## 2. Methods

### 2.1. Materials

The *Pichia pastoris* cell expression system including the pPicZα plasmid was obtained from the EasySelect™ Pichia Expression Kit (Invitrogen, Carlsbad, CA, USA: K174001). For immunoblotting, the primary antibody was c-Myc Monoclonal Antibody (9E10) (Invitrogen, Carlsbad, CA, USA: 13-2500), and the secondary antibody was Goat anti-Mouse IgG, IgM (H+L) Cross-Adsorbed Secondary Antibody, AP (Invitrogen, Carlsbad, CA, USA: 31330).

### 2.2. Cloning, Verification, and Transformation of Cp3GT into Pichia pastoris

A pPicZα plasmid https://www.snapgene.com/plasmids/yeast_plasmids/pPICZ_A (accessed on 10 November 2023) containing the Cp3GT coding region (GQ141630) was transformed into *Escherichia coli* cells for propagation and grown as previously described [49]. After cultivation, plasmid DNA was isolated and sequenced to confirm the wild-type Cp3GT gene’s presence, as previously demonstrated [49]. Using site-directed mutagenesis, a thrombin cleavage sequence (LVPRGS) was integrated upstream of the C-terminal c-myc/6x His recombinant tags, to allow post-purification tag removal (Supplemental Figure S1).

This modified plasmid was subsequently introduced into competent *Pichia pastoris* cells via electroporation, as previously described [49]. To confirm the Cp3GT gene’s successful integration into the *Pichia* genome, colony PCR was performed using 5′ and 3′ primers for the plasmid-encoded alcohol oxidase (AOX) gene. DNA sequencing from PCR-positive colonies confirmed the correct integration of the Cp3GT gene with the thrombin cleavage sequence [49].

### 2.3. SDS-PAGE

The protein samples were prepared for SDS-PAGE by mixing in a 1:1 ratio with Laemmli loading buffer containing 5% β-mercaptoethanol (BME). The mixture was then boiled for 5 min to ensure denaturation. The samples were loaded onto a 10% polyacrylamide gel and electrophoresed at 100 V for approximately 1 h using 1× Tris-Glycine-SDS running buffer. The composition of the running buffer was as follows: 25 mM Tris, 192 mM Glycine, and 0.1% SDS.

One gel was stained using Coomassie Blue staining solution (0.1% Coomassie Blue R250 in 10% acetic acid, 50% methanol, and 40% water). Post-staining, the gel was de-stained using a solution comprising 10% glacial acetic acid, 20% methanol, and 70% distilled water.

A duplicate gel was used for immunoblotting. The proteins were transferred onto a nitrocellulose membrane in a transfer buffer (1× Tris-Glycine containing 20% methanol; composition: 25 mM Tris, 192 mM Glycine). The membrane was then blocked using a blocking buffer composed of 5% non-fat milk powder in 1× TBST (20 mM Tris-HCl, 150 mM NaCl, 0.1% Tween 20, pH 7.4), with the addition of 0.1% sodium azide. The blocking was carried out for 2 h at room temperature.

Post-blocking, the membrane was rinsed with TBST and incubated with an anti-C-myc mouse monoclonal antibody, diluted 1:2500 in TBST, for 2 h. Following primary antibody incubation, the membrane was washed three times with TBST for 5 min each time. The membrane was then incubated with a goat anti-mouse IgG conjugated alkaline phosphatase diluted 1:10,000 in TBST, for 2 h. This was followed by another set of washes in TBST, three times for 5 min each., 

For detection, the immunoblot was developed using alkaline phosphatase substrate. This was achieved by dissolving a SIGMAFAST™ BCIP^®^/NBT tablet (Sigma-Aldrich, Burlington, MA, USA: B5655) in 10 mL of distilled water and by applying the solution to the membrane. The development was monitored and stopped after approximately 2 min when bands appeared.

For gels of the samples with low protein content (less than 0.3 µg), silver staining was conducted using the Pierce™ Silver Stain Kit (Thermo Scientific, Waltham, MA, USA: 24612), following the manufacturer’s instructions. The development of silver stains was halted after approximately 2 min, once the bands were visible.

### 2.4. Pichia pastoris Growth and Expression Screening

The growth curve and expression profile of transformed *P. pastoris* were determined by inducing with methanol at 28 °C, as described previously [49], with samples taken at 4, 8, 21, 25, 29, 33, 45, and 72 h intervals. The samples at each time point were analyzed for absorbance at an optical density (OD) of 600 nm. At each time point, 2 mL of culture was centrifuged at 1500× *g* for 5 min at 4 °C, resuspended in 200 µL cell lysis buffer (50 mM NaPO_4_, 1 mM PMSF, 5 mM βME, 5% glycerol, pH 7.4), and lysed using glass beads with vortexing at high speed. The lysed samples were centrifuged at max speed for 10 min at 4 °C. The supernatant was transferred to a new tube, and the crude protein concentration was estimated using absorbance at 280 nm. For each time point, 100 µg of crude protein was analyzed by SDS-PAGE and immunoblot.

### 2.5. Experimental Scale-Up and Lysis

The following parameters were experimentally determined to be the most efficient for larger-scale culture, expression, and purification. Transformed *P. pastoris* clones with recombinant Cp3GT were streaked on yeast peptone dextrose (YPD) agar and incubated at 28 °C for 3 days. Single colonies from these clones were inoculated into four respective 5 mL buffered minimal glycerol yeast media (BMGY) aliquots, followed by incubation at 28 °C, with shaking at 250 rpm for 24 h. From each of the four cultures, 1 mL was used to inoculate 100 mL BMGY aliquots in 250 mL baffled flasks. These aliquots were then incubated at 28 °C, with shaking at 250 rpm for another 24 h. From each flask, 100 mL of cell culture was collected, centrifuged at 1500× *g*, and then resuspended in 1 L aliquots of buffered minimal methanol (0.5%) yeast media (BMMY) to achieve a final OD_600_ of 1. These resuspended cells were placed into 3 L baffled flasks and incubated for 24 h at 28 °C, with shaking at 250 rpm. The cells were then harvested by centrifugation at 1500× *g* at 4 °C. The combined 4 L cell pellet was then resuspended in 100 mL of cell lysis buffer and lysed using a ThermoSpectronic French Pressure Cell (Conquer Scientific, San Diego, CA, USA: FA-078) at 20,000 PSI for 5 cycles at 4 °C. The lysate was centrifuged at 20,000× *g* for 20 min at 4 °C. The supernatant was transferred to a new vessel and PMSF was added to a final concentration of 1 mM.

### 2.6. Cp3GT Cobalt Affinity Chromatography

Prior to scaling up, the cell lysate was transitioned into equilibration buffer (50 mM NaPO_4_, 300 mM NaCl, 10 mM Imidazole, 5 mM βME pH 7.4) using PD-10 prepacked columns. The lysate was then evenly distributed across 12 3.25 mL His-Pur Cobalt Resin (Thermo Scientific, Waltham, MA, USA: 89964) aliquots in gravity flow columns, which were previously equilibrated in equilibration buffer. After applying the lysate, each column was washed with 200 mL of wash buffer (50 mM NaPO_4_, 300 mM NaCl, 20 mM Imidazole, pH 7.4) in 50 mL intervals. A small sample was collected after every 50 mL interval for analysis. Cp3GT was eluted in 10 mL of elution buffer (50 mM NaPO_4_, 250 mM Imidazole, pH 7.4) in 1 mL fractions. Crude lysate, flowthrough, wash, and eluate were analyzed using SDS-PAGE and immunoblot.

### 2.7. Cp3GT Anion Exchange and Size Exclusion Chromatography

For anion exchange chromatography, MonoQ resin was selected due to its high resolution and compatibility with the protein of interest. The initial optimization trials involved varying the NaCl gradient concentration from 0 to 1 M over 30 mL to identify the ideal elution profile. The flow rate was maintained at 0.5 mL/min to balance between resolution and time efficiency. The choice of buffer composition, primarily 25 mM Bicine with varying NaCl concentrations, was based on preliminary tests that indicated the optimal protein stability and minimal nonspecific interactions under these conditions.

The cobalt affinity eluate fractions containing Cp3GT were collected. A 500 µL sample was dialyzed to remove imidazole and promptly tested for GT activity, followed by visualization using SDS-PAGE and immunoblot. The remaining eluate was placed into an Amicon Ultra-15 centrifugal concentrator (Millipore, Burlington, MA, USA: UFC9030) and concentrated according to the manufacturer’s instructions. Buffer A (25 mM Bicine, 14 mM βME pH 8.5) was added to the centrifugal concentrator to a final volume of 15 mL. The eluate was again concentrated. This process was repeated two more times to ensure that the cobalt affinity elution buffer was completely exchanged into Buffer A.

A 5 mL sample of concentrated, desalted His-Pur eluate was further enriched by a Fast Protein Liquid Chromatography (GE ÄKTA Purifier; 10 mL Superloop on a MonoQ 5/50 GL (Cytiva 17516601) prepacked column. Initial column loading was at a flow rate of 0.5 mL/min with the flowthrough collected in 5 mL volumes. Cp3GT was eluted using a 20% gradient of Buffer B (25 mM Bicine, 1 M NaCl, 5 mM βME) for 48 min in 0.5 mL volumes at a flow rate of 0.5 mL/min. The flowthrough and all peaks showing absorbance were assayed for activity and analyzed using SDS-PAGE and immunoblot.

The MonoQ fractions containing Cp3GT were combined and concentrated to a final volume of 150 µL in Buffer A. This sample was then fractionated on a Superdex 75 10/300 GL (Cytiva, Muskegon, MI, USA: 17-5174-01) prepacked FPLC column, which had been previously equilibrated with MonoQ Buffer A at a flow rate of 0.5 mL/min. The selection of Sephadex 75 was based on its separation range, which was apt for proteins in the molecular weight range of Cp3GT and for the removal of the larger contaminant. This column was effective in distinguishing Cp3GT from contaminant proteins with a higher molecular weight; thus, it enhanced the purity of the sample. Its established efficacy in separating proteins within this specific range was a key determinant for its utilization. The resulting peaks were analyzed using SDS-PAGE and immunoblot.

### 2.8. Cp3GT Glucosyltransferase Activity Assay

Glucosyltransferase activity was assayed by measuring the incorporation of a radiolabeled ^14^C-Glucose, as previously described [50,51]. Briefly, a 75 µL reaction was prepared by mixing 5 µL of quercetin aglycone (50 nmol/5 µL ethylene glycol monomethyl ether), 10 µL of 100 nmol UDP-^14^C-glucose containing 40,000 cpm, 5–15 µL of purified enzyme (added upon starting), and enough 50 mM phosphate buffer (pH 7.5 containing 14 mM βME) to make a total reaction volume of 75 µL [50]. The reactions were run for 5–45 min at 37 °C and stopped with 10 µL of 6 M HCl. The glucosylated product was extracted by adding 250 µL ethyl acetate, and the reaction mix was vortexed for 20 s, followed by microcentrifugation at max speed for 10 s. From the top layer, 100 µL was taken and added to 2.5 mL CytoScint Liquid Scintillation Cocktail (MP Biomedicals, Santa Ana, CA, USA: 882453) and counted for 2 min in a Beckman Coulter LS 6500 Scintillation Counter. The glucosylated product amount in nanomoles was determined by multiplying the counts per minute (cpm) by 2.5 to correct for volume and then divided by 400 (400 cpm/nmol UDP-G).

To quickly assess the GT activity in the column fractions, a screening assay was implemented as previously described [50], with the following exceptions: 10 µL UDP-14C glucose (containing 20,000 cpm) was taken directly from a source vial with no other UDP-G added and 2–20 µL of the column fraction was used. The reactions were conducted for 2–5 min.

### 2.9. Model Generation and Ligand Binding Site Prediction

Cp3GT models were generated using the Distance-Guided Protein Structure Prediction (D-I-TASSER) server “https://zhanggroup.org/D-I-TASSER/” (accessed on 15 November 2023) [37]. Options were selected to predict protein function based on structural models, utilize the large IMG/JGI metagenomic database for multiple sequence alignment (MSA) construction, and implement the integrated AlphaFold2 pipeline. Models were ranked based on their TM-scores, and the model with the highest-ranking score was selected for structural analysis.

All the ligand binding site predictions were generated using the D-I-TASSER-integrated COFACTOR algorithm “https://zhanggroup.org/COFACTOR/” (accessed on 15 November 2023) [52,53]. The highest-ranking template that included a flavonol ligand, preferably quercetin or kaempferol, was chosen for analysis. Structural alignments were generated using UCSF Chimera “https://www.cgl.ucsf.edu/chimera/” (accessed on 12 November 2023) [54]. The models were analyzed using Biovia Discovery Studio [55] and Maestro [56].

## 3. Results and Discussion

### 3.1. Growth and Expression Analysis

During induction, *P. pastoris* exhibited exponential (or logarithmic) growth for the initial 20 h, after which it entered the stationary phase and the growth plateaued (Supplemental Figure S2A). Cp3GT expression was detected at 4 h and continued to increase until 25 h, after which no increase in expression was detected (Supplemental Figure S2B,C). A band with the predicted size of Cp3GT (65.6 kDa) was identified upon recombinant expression, and it increased with expression time. A band approximating 56.6 kDa emerged faintly at 20 h, intensifying until it peaked around 45 h. Due to concerns about Cp3GT degradation, the subsequent cultures were limited to a 20 h growth duration.

The exponential growth phase during the first 20 h signifies a robust metabolic state for the yeast, providing an ideal cellular environment for recombinant protein expression. Notably, the peaking of the Cp3GT expression at around 25 h aligns well with the transition of the yeast cells from the exponential to the stationary phase. This suggests an optimal window for recombinant protein synthesis before the cells divert resources in response to changing growth conditions. The presence of a 56.6 kDa band, appearing later in the expression timeline, could be indicative of proteolytic degradation, which warranted further investigation. The decision to restrict culture growth to 20 h for subsequent experiments seems prudent given this potential degradation, as it capitalizes on the optimal expression window while reducing the chances of unwanted protein modifications or breakdown.

### 3.2. Cp3GT Cobalt Affinity Chromatography Analysis

In the scale-up experiments for Cp3GT purification, the optimal loading capacity was determined to be 2.5 mL of affinity resin for 250 mL of lysed culture. To avoid the bottlenecks associated with PD-10 desalting columns and to reduce Cp3GT degradation, the cell lysate supernatant was diluted 10-fold with equilibration buffer after centrifugation, enhancing the purification process.

A total of 40 mL of affinity resin was employed for the purifying protein from 4 L of cell culture, distributed across 12 columns in 3.25 mL aliquots to minimize nonspecific binding and to maintain flow rates. Coomassie staining and immunoblot analysis confirmed the presence of Cp3GT in the crude lysate, with no detection in the flowthrough or wash fractions (Supplemental Figure S3A,C). Cp3GT elution was observed in fractions 4–7, along with multiple bands in the eluate, including a distinct 100 kDa protein (Supplemental Figure S3B,D).

The detection of an additional band in the eluate, similar in size to Cp3GT (Supplemental Figures S2 and S3B,D), hinted at a Cp3GT degradation product containing a c-myc/6x His tag. This finding suggests N-terminal degradation and highlights the importance of maintaining protein integrity during purification. The use of PMSF effectively reduced this degradation. The cobalt affinity purification method, while successful as an initial step, necessitated further chromatographic steps for optimal purity [34,35,36,57].

### 3.3. Anion Exchange Chromatography

In the pursuit of enhancing Cp3GT purification, anion exchange chromatography using MonoQ resin was employed, capitalizing on its track record in purifying plant GTs [50,58,59]. The affinity eluate underwent MonoQ chromatography, yielding four primary peaks across fractions 8–11, identified at varying NaCl concentrations (Supplemental Figure S4A). Notably, Peak 4 displayed two bands differing by approximately 9 kDa (Supplemental Figure S4B), suggesting N-terminal degradation and a potential shift in the protein’s isoelectric point (pI) and anionic potential. This was corroborated by immunoblot analysis, which showed distinct bands at 65 and 56 kDa (Supplemental Figure S4C).

It was hypothesized that degradation during Cp3GT’s purification resulted in the loss of approximately 9 kDa at the N terminus, which is equivalent to around 80 amino acids and would change its theoretical pI from 6.38 to 5.92. Such a change might affect the protein’s anionic potential, influencing its binding affinity to the column and causing it to elute at higher NaCl concentrations (Supplemental Figure S4). It is also possible that the combination of high cell density from the scale-up and PMSF’s instability in aqueous solutions was leading to insufficient inhibition of the endogenous proteases. To address this, subsequent purifications introduced PMSF not only during cell lysis but also after centrifugation of the lysed cells. By adding PMSF to the supernatant just before loading it onto the affinity column, the emergence of the second band was markedly reduced.

The employment of anion exchange chromatography using MonoQ demonstrated mixed outcomes in the pursuit of Cp3GT purification. The observation of different elution peaks underpins the intricate and dynamic nature of protein purification. As discussed previously, the detection of Cp3GT at varying sizes, as evidenced by the immunoblot, raises an inherent question about the protein’s structural integrity during the purification process. The 9 kDa difference, estimated to correspond to approximately 80 amino acids, indicates a degradation event. Given the revised theoretical pI of 5.92 for the truncated protein, one can infer that the change in pI due to N-terminal truncation might have implications for the protein’s interaction with the anionic column, leading to a shift in its elution profile. This is shown by an increased presence of this degradation band eluting at slightly higher salt concentrations.

Furthermore, the possibility of inadequate protease inhibition, especially with PMSF’s instability in aqueous solutions, becomes a pressing concern. The practice of adding PMSF post-centrifugation was performed as a response to these challenges. Its apparent efficacy in curbing the emergence of the second band may point to the broader necessity for dynamic adjustments in protein purification workflows, especially when handling labile or degradation-prone proteins.

### 3.4. Anion Exchange Chromatography: Refining the Gradient and Verifying Cp3GT Integrity

In a refined purification process, the immunoblotting of both the crude and the affinity-purified eluates revealed two distinct bands at 65 and 56 kDa, with the 65 kDa band demonstrating increased prominence compared to the earlier results (Supplemental Figure S6). This was further confirmed as the 56 kDa band, which is indicative of Cp3GT degradation, was absent in the MonoQ fractions. The affinity eluate also exhibited a pronounced 100 kDa band (Supplemental Figure S6).

For enhanced resolution, a shallower gradient of 20% Buffer B over 50 mL was utilized in MonoQ chromatography (Supplemental Figure S5). This approach effectively separated three key peaks at fractions 14, 16, and 18, corresponding to different NaCl concentrations. These peaks were subjected to GT activity assays and analyzed through SDS-PAGE and immunoblotting (Supplemental Figure S6).

**Figure 1:**
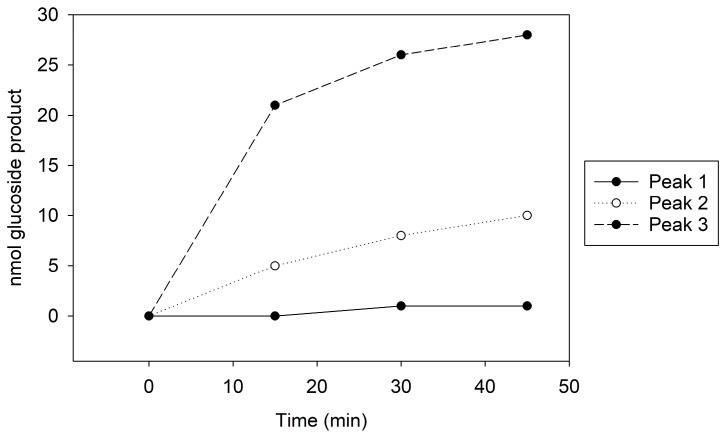
MonoQ Peaks 1–3 GT activity assay time course. Assay conducted using 10 µL of each sample for 45 min.

Peak 1, which showed no GT activity (Figure 1), presented a singular 100 kDa band on the Coomassie stain and a faint 65 kDa band on the immunoblot (Supplemental Figure S6A,B). In contrast, Peak 2, with moderate GT activity (Figure 1), revealed both 100 kDa and 65 kDa bands on the Coomassie stain and a distinct 65 kDa band on the immunoblot (Supplemental Figure S6A,B). Peak 3, exhibiting the highest GT activity (Figure 1), similarly displayed 100 kDa and 65 kDa bands on Coomassie, but the immunoblot indicated a more intense 65 kDa band compared to the other peaks (Supplemental Figure S6A,B). The kinetic assay and immunoblot results confirmed the identity of the 65 kDa protein as Cp3GT.

Optimizing the MonoQ gradient significantly improved the resolution of the Cp3GT peaks. The simultaneous observation of two bands at 65 and 56 kDa in both the crude and affinity-purified eluates pointed towards Cp3GT degradation, but the enhanced intensity of the 65 kDa band suggested better enzyme integrity. Notably, the 56 kDa band was absent in the MonoQ fractions, while a 100 kDa band consistently co-eluted with Cp3GT, necessitating further investigation into its role and interaction with Cp3GT.

Employing a shallower gradient led to a more nuanced separation of Cp3GT peaks, reinforcing the importance of fine-tuning the chromatographic conditions. The consistent detection of the 65 kDa band across peaks, with the highest GT activity peak showing an intensified 65 kDa band on the immunoblot, confirmed its identity as intact Cp3GT (Supplemental Figure S7). The presence of the 100 kDa protein across all the peaks, which was particularly strong in Peak 1, remained an enigma.

**Figure 2:**
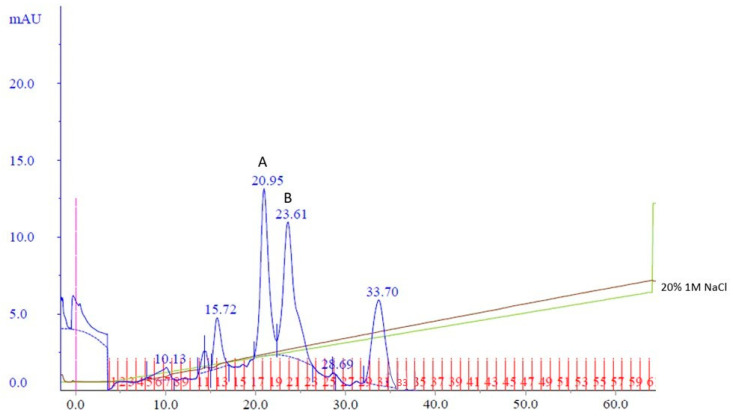
MonoQ anion exchange chromatography of cobalt affinity purified Cp3GT eluate #3. Two peaks of interest labeled A and B, eluted at 72 and 83 mM NaCl, respectively, using a 20% gradient of Buffer B over 60 mL. Green line represents NaCl gradient. Brown line represents conductivity. Blue lines represent absorbance. Blue numbers represent elution volume at specified peak. Pink line represents sample injection point. Red lines and numbers indicate fraction numbers.

**Figure 3:**
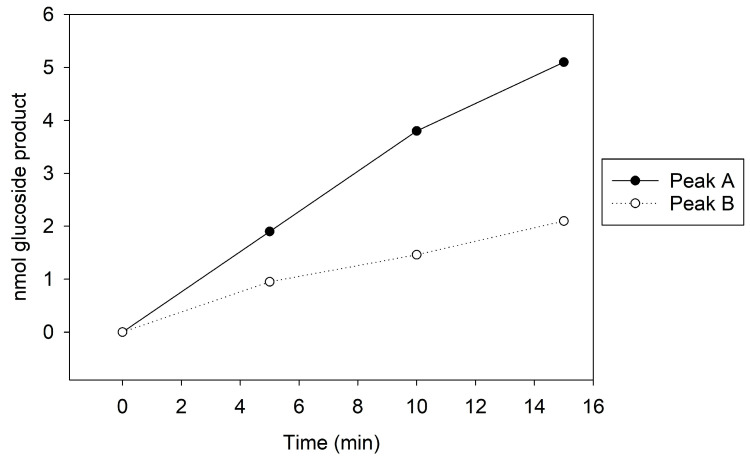
MonoQ Peaks A and B GT activity assay time course. Assay conducted using 5 µL of sample for 15 min.

To potentially separate Cp3GT from the 100 kDa protein, a gentler elution gradient of 20% Buffer B over 60 mL was applied. This resulted in two distinct peaks, Peak A and Peak B, at 72 and 83 mM NaCl, respectively (Figure 2). Peak A demonstrated higher GT activity than Peak B (Figure 3). Immunoblotting revealed a 65 kDa band in Peak A, identified as Cp3GT, and a slightly smaller 64 kDa band in Peak B, which was likely a mildly degraded form of Cp3GT with minor N-terminal loss (Supplemental Figure S7). The 64 kDa band’s retention of GT activity and positive immunoblot results suggest its close association with intact Cp3GT, despite potential minor degradation. The potential overlap between Peaks A and B might also contribute to the observed activity in Peak B (Figure 2).

Coomassie staining revealed a distinct pattern across two peaks in the MonoQ chromatography. Initially, a 100 kDa protein eluted separately in fraction 17 and subsequently co-eluted with Cp3GT, diminishing in intensity up to fraction 20. In fraction 21, Cp3GT eluted independently, without the 100 kDa contaminant (Supplemental Figure S7). GT kinetic assays and immunoblotting confirmed the presence of Cp3GT in Peak A. The 56 kDa degradation product, observed in both the crude and affinity eluates, was absent in the MonoQ elutions, indicating the effectiveness of additional PMSF in reducing degradation (Supplemental Figure S7).

The use of a more gradual NaCl gradient in MonoQ chromatography enhanced the separation of Cp3GT from the 100 kDa protein, as seen by the distinct elution patterns. The appearance of a slightly smaller 64 kDa band in Peak B, alongside GT activity and immunoblot identification, suggests it is a minorly degraded form of Cp3GT, possibly due to partial N-terminal degradation or protein overlap from Peak A.

While the focus of the study was to purify Cp3GT, the distinct elution pattern, especially the separate elution of the 100 kDa protein, offers insights into potential interactions or affinities between Cp3GT and this protein. Future research may explore the nature of this relationship further. The consistent absence of the 56 kDa degradation product in MonoQ chromatography, following the introduction of PMSF, highlights the importance of this step in maintaining Cp3GT’s stability and integrity during purification.

### 3.5. Cell Handling and Storage Modifications Reduce Cp3GT Degradation and Further Refine Anion Exchange Chromatography

To mitigate the Cp3GT degradation risks, the cell handling methods were modified. The cells were lysed immediately post-induction and centrifuged to separate the debris. The supernatant was promptly treated with 1 mM PMSF and 20% glycerol, then stored at −80 °C, reducing the Cp3GT’s exposure to proteases and enhancing its stability.

**Figure 4:**
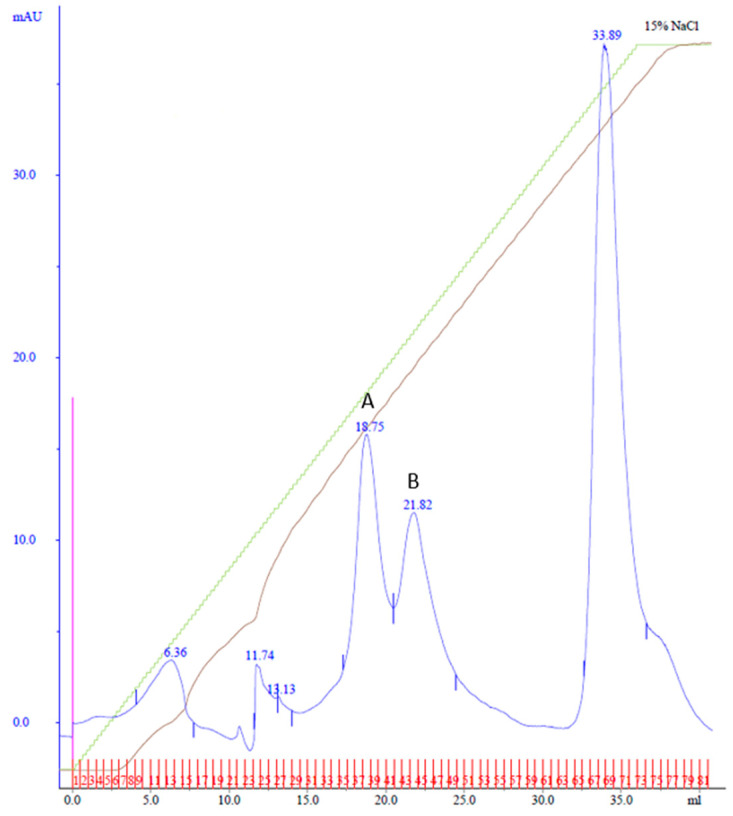
MonoQ anion exchange chromatography of cobalt affinity purified Cp3GT eluate #4. Two peaks of interest labeled A and B, eluted at 78 and 90 mM NaCl, respectively, using a 15% gradient over 37 mL. Green line represents NaCl gradient. Brown line represents conductivity. Blue lines represent absorbance. Blue numbers represent elution volume at specified peak. Pink line represents sample injection point. Red lines and numbers indicate fraction numbers.

Purification using affinity and MonoQ chromatography followed. In MonoQ, a 15% Buffer B gradient over 37 mL was used, and the fractions were collected in 0.5 mL increments. This yielded two distinct peaks: Peak A, eluting between fractions 34 and 40 at 78 mM NaCl, and Peak B, eluting across fractions 41–48 at 90 mM NaCl (Figure 4). The shallower gradient enhanced the separation and resolution of the protein peaks.

Following cell handling adjustments, the fractions from the MonoQ chromatography were tested for GT activity and analyzed via SDS-PAGE (Supplemental Figure S8). GT activity was confirmed in Peaks A and B. The 100 kDa band, observed in previous purifications, eluted just before Cp3GT in both peaks (Supplemental Figure S8). Notably, the 56 kDa degradation product was absent throughout, indicating enhanced Cp3GT stability with this method.

These results underscored the importance of immediate cell lysis, PMSF, and glycerol application post-induction and of avoiding cell freezing before lysis. The emergence of the distinct Peaks A and B suggests two differently charged Cp3GT forms under these conditions. The consistent presence of the 100 kDa band and its relationship with Cp3GT remain to be explored. This study’s findings lead to potential improvements in purification techniques, including the use of a gradual gradient in MonoQ chromatography and the consideration of gel filtration as a final purification step.

### 3.6. Cp3GT Size Exclusion Chromatography Analysis

It was hypothesized that Cp3GT could be effectively separated from the 100 kDa contaminant protein using size exclusion chromatography. To test this hypothesis, all the MonoQ elution fractions containing Cp3GT were pooled, concentrated, and applied to a Superdex 75 size exclusion column. The aim of this strategy was to purify Cp3GT to apparent homogeneity, setting the stage for future crystallographic and other structural analyses.

**Figure 5:**
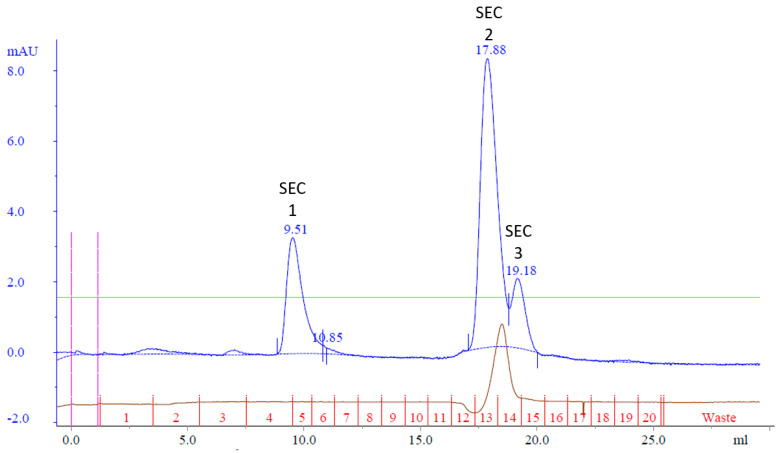
Size exclusion chromatography of MonoQ purified Cp3GT: three peaks of interest labeled SEC 1−3 were analyzed for separation of Cp3GT from 100 kDa protein. Green line represents NaCl gradient. Brown line represents conductivity. Blue lines represent absorbance. Blue numbers represent elution volume at specified peak. Pink line represents sample injection point. Red lines and numbers indicate fraction numbers.

**Figure 6:**
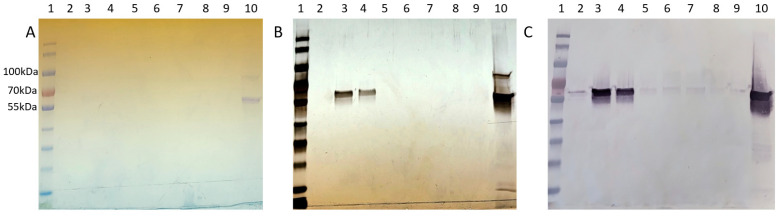
Cp3GT size exclusion chromatography profile: (**A**) Coomassie stain, (**B**) silver stain, and (**C**) immunoblot of Superdex 75 purification loaded with 1. MWM, 2−4. SEC Peak 1 in fractions 4−6, 5. Peak 2 (fraction 12), 6. Peak 2 (fraction 13), 7. Peak 2/3 (fraction 14), 8. Peak 3 (fraction 15), 9. Loading dye, 10. Pooled MonoQ.

The resulting chromatogram revealed three distinct peaks, labeled SEC Peak 1–3 (Figure 5). Coomassie staining faintly detected Cp3GT in Peak 1; however, it was more visible by silver stain and immunoblot (Figure 6). Notably, despite their stronger absorbance, no proteins were detected by Coomassie in Peaks 2 and 3; however, there were faint bands detected using immunoblot (Figure 6). The 100 kDa contaminant was absent, suggesting its elution prior to Cp3GT. The fractions before the first peak, showing low absorbance, were not analyzed.

The disparity in detection between Coomassie stain, silver stain, and Western blotting is noteworthy. Coomassie Blue, a commonly used protein stain, is less sensitive and can typically detect proteins in the range of 0.1 to 1 µg. In contrast, silver staining is more sensitive, with detection limits as low as a few ng of protein, making it suitable for visualizing proteins present in lower concentrations. Western blotting, while specific to the protein of interest, is influenced by the affinity of the antibody and can detect even lower amounts of protein compared to silver stain. The observed differences in detection between these methods are consistent with their respective sensitivities and specificities. The variation in staining intensity and detection across these methods highlights the differential abundance of Cp3GT and its variants in the size exclusion fractions, underlining the importance of employing multiple detection techniques for a comprehensive analysis of protein purification.

Size exclusion chromatography, given its reliance on molecular size for separation, was an apt choice to address the persistent issue of coelution involving the 100 kDa protein. By employing a Superdex 75 size exclusion column, it was anticipated that Cp3GT could separate from the 100 kDa contaminant protein. The observed absence of this 100 kDa band in the resulting fractions corroborated the strength of this strategy.

The chromatogram obtained, with its three discernible peaks, points toward a complexity of proteins or protein states in the sample. The detection disparity between Coomassie and silver staining, as well as immunoblotting, indicates differences in the Cp3GT concentration in the elution fractions. Notably, the detection of pure Cp3GT in SEC Peak 1 is notable. This outcome, combined with the absence of the 100 kDa contaminant, underscores the significance of size exclusion chromatography in the purification toolkit.

### 3.7. Purification Procedure Summary

Cp3GT was successfully expressed in *P. pastoris*, and the procedure was fine-tuned to consistently yield recombinant Cp3GT devoid of degradation. Through meticulous development and assessment, Cp3GT was efficiently purified to apparent homogeneity utilizing a composite chromatographic approach that harnessed the strengths of affinity, anion exchange, and size exclusion chromatography. This methodical purification strategy ensures the reproducible production of intact, homogenous Cp3GT.

Achieving highly pure and intact recombinant Cp3GT is particularly significant in the context of advanced structural analyses. Direct experimental methods, such as X-ray crystallography and cryogenic electron microscopy, demand elevated concentrations of unadulterated protein to render meaningful outcomes. With a consistent source of pure Cp3GT now available, the stage is set for the employment of these techniques to delve deeper into the protein’s structural dynamics.

### 3.8. Cp3GT, Cp3GTΔ10, and Cp3GTΔ80 Structure Generation and Ligand Binding Site Prediction

Recent advancements in protein structural prediction tools that incorporate deep learning and machine learning algorithms offer an avenue for more intricate understanding of protein–ligand interactions. Given this context, it was hypothesized that an in-depth examination of Cp3GT structure and the interactions between Cp3GT residues and its flavonol substrates could be achieved through updated structural prediction models. D-I TASSER was selected for this analysis due to its notable accuracy in CASP15, its integration with the AlphaFold 2 pipeline, and its capability to incorporate ligands and generate docking poses using COFACTOR [37].

The persistent presence of degradation products during the purification process motivated a comparison of the intact Cp3GT, equipped with C-terminal c-myc/6x His recombinant tags and a thrombin cleavage sequence [31,49], with a truncated variant that was devoid of the initial 80 amino acids on the N-terminus (Cp3GTΔ80) through in silico modeling. Additionally, a variant of Cp3GT with a truncation of the first 10 amino acids on the N-terminus (Cp3GTΔ10) was introduced into the analysis due to its demonstrably altered activity (Figure 3). The decision to study the 80-amino-acid truncation was driven by an observable size discrepancy of approximately 9 kDa, corresponding to about 80 amino acids, as evidenced in the Coomassie and immunoblot analyses (Figure 1, Figure 2 and Figure 3). The rationale behind examining the 10-amino-acid truncation stems from the size difference of roughly 1–1.5 kDa (Figure 4) or, equivalently, around 10 amino acids.

In this endeavor, the primary aim was to elucidate new insights into Cp3GT structural characteristics that give rise to activity as well as flavonol specificity and assess how degradation events might impact Cp3GT structure and activity using the most recent, technologically advanced in silico tools. Additionally, this research aimed to validate the accuracy of these structural models and ligand binding site predictions by comparing them with an anthocyanidin/flavonol-specific GT (VvGT1) with 59% homology with Cp3GT [34], crosschecking them with previous docking experiments that used Autodock [32,33] and evaluating the models in the context of previous biochemical analyses of Cp3GT with mutated residues.

The highest-ranking models generated by D-I-TASSER were used for analysis, with the estimated TM-scores for Cp3GT, Cp3GTΔ10, and Cp3GTΔ80 being 0.63, 0.64, and 0.61, respectively. These scores were considered acceptable for analysis; however, the inclusion of C-terminal recombinant tags in the modeling resulted in lower scores because the reference PDB crystal structures used as templates did not report such tags. Indeed, when Cp3GT was modeled without tags, the TM-scores for Cp3GT, Cp3GTΔ10, and Cp3GTΔ80 were 0.65, 0.65, and 0.66, respectively (Supplemental Figures S9–S11).

The D-I-TASSER pipeline subjected each model to the COFACTOR algorithm and produced the scoring parameters seen in Table 1. Cp3GT modeled without recombinant tags was also processed through COFACTOR and generated slightly higher confidence values (Supplemental Table S1). The analysis of these data provided insights into the binding characteristics of the Cp3GT variants. All three protein models, Cp3GT, Cp3GTΔ10, and Cp3GTΔ80, displayed remarkably consistent C-scores of approximately 0.34. This uniformity suggested a comparable level of confidence in the ligand binding predictions, despite the N-terminal truncations.

Each prediction’s foundation on the structure of VvGT1, as evidenced by the PDB analog, was noteworthy. Given VvGT1’s established role in flavonoid glucosylation and its solved crystal structure, its selection as a reference underscored its relevance to the current study [34]. Furthermore, the TM-scores for all the models surpassed the 0.8 threshold, which was indicative of a close structural relationship with the VvGT1 reference. Complementing this, the RMSD values were uniformly proximate to 1.4, suggesting a consistent and accurate prediction of the atomic positions.

The sequence similarity, with IDEN values around 0.6, implied a moderate to high resemblance between the proteins in question and VvGT1. Such congruence might have influenced the server’s choice of VvGT1 as a template and impacted the predicted ligand binding sites of the proteins. An equally significant observation was the coverage value. With values close to 0.87, it was evident that much of the protein structure found alignment with the reference, suggesting strong modeling accuracy.

The focus on quercetin and kaempferol aligned perfectly with the emphasis on acceptor flavonol substrates. Their presence in the predictions further accentuated the functional significance of these molecules for Cp3GT. The BS-score, a measure reflecting the local similarity between template and predicted binding sites, offered yet another layer of depth. All models had scores above 1, suggesting a significant match between the predicted and template binding sites and instilling greater confidence in the binding predictions provided.

### 3.9. Structure Differences and Similarities of Cp3GT with Cp3GTΔ10 and Cp3GTΔ80

In this study, the truncated Cp3GT variants, Cp3GTΔ10 and Cp3GTΔ80, were investigated using in silico approaches to understand the potential structural changes and their impact on GT activity. These variants were not physically produced in vivo; rather, they were conceptualized and analyzed through computational methods. The comparative structural analysis of intact Cp3GT with the in silico models of Cp3GTΔ10 and Cp3GTΔ80 aimed to elucidate the structural deviations and their functional implications.

The superimposition of Cp3GT and Cp3GTΔ10 indicated minimal structural deviations and did not immediately elucidate the observed decrease in GT activity. While the subtle disparities between these structures did not directly illuminate the diminished GT activity, a conceivable explanation might involve the overlap of the Peak A residual in Peak B, warranting further investigation into whether the truncation allows folding that retains some level of activity, albeit reduced. The detailed discussion on Cp3GTΔ10 is further elaborated in subsequent sections; here, the focus on Cp3GTΔ80 is maintained.

**Figure 7:**
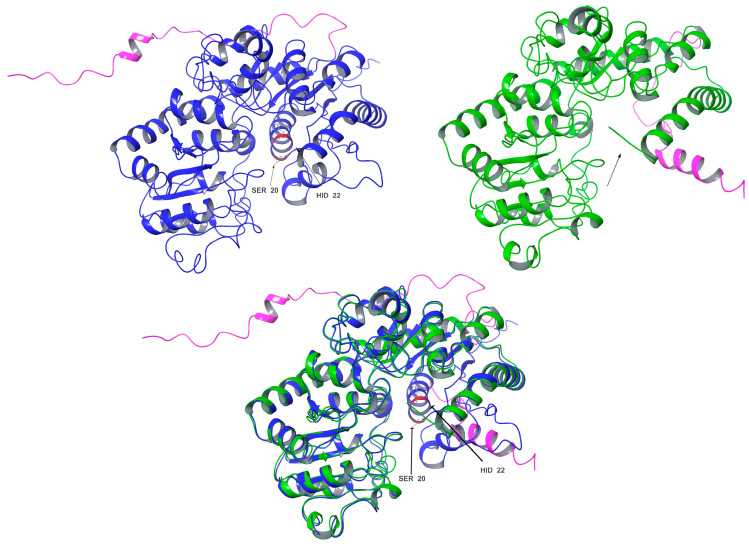
Three-dimensional structural comparison of Cp3GT with an N-terminal truncated variant Cp3GTΔ80. Cp3GT (**top left**) in blue and Cp3GTΔ80 (**top right**) in green showing C-myc tag/6x His in pink. Black arrows point to the alpha helix in the catalytic cleft containing catalytic residues HID-22 and SER-20 (red). The alpha helix is notably absent in Cp3GTΔ80. Superimposition of Cp3GT with Cp3GTΔ80 (**bottom center**) highlights structural differences between the two.

In contrast, the comparisons between Cp3GT and Cp3GTΔ80 indicated clear and significant structural alterations that could have implications for the enzyme’s activity. Notably, the absence of an alpha helix within the catalytic cleft of Cp3GTΔ80, which contains the critical residues HID-22 and SER-20, underlines the potential for this truncation to result in a protein inactivation. The HID designation signifies that the hydrogen is attached to the δ-nitrogen (Nδ), which is bonded to the carbon in the double bond of the imidazole ring. Furthermore, the docking residues GLN-87, HID-154, GLU-192, and PHE-203 downstream of the 80-amino-acid deletion were conserved in their orientation in both models (Figure 7).

Importantly, the conclusion drawn from these data is that Cp3GTΔ80 lacks the ability to catalyze flavonol 3-O glucosylation, primarily due to the absence of HID-22. This amino acid is a universally conserved catalytic residue and is crucial for maintaining the functional integrity of GTs.

### 3.10. Distinct Binding Dynamics in the Cp3GTΔ80-Kaempferol Model

The ligand binding analysis for Cp3GTΔ80 was conducted using kaempferol as the substrate. This choice was driven by COFACTOR’s prediction, which selected kaempferol as the binding template for Cp3GTΔ80, whereas it favored quercetin for Cp3GT and Cp3GTΔ10. While Cp3GT biochemical characterization has repeatedly shown quercetin to be the preferred substrate, the enzyme’s capability to glucosylate kaempferol ensures the relevance of this analysis [30,31,32,33]. It is noted that the 80-amino-acid deletion in Cp3GTΔ80 altered COFACTOR’s substrate prediction.

**Figure 8:**
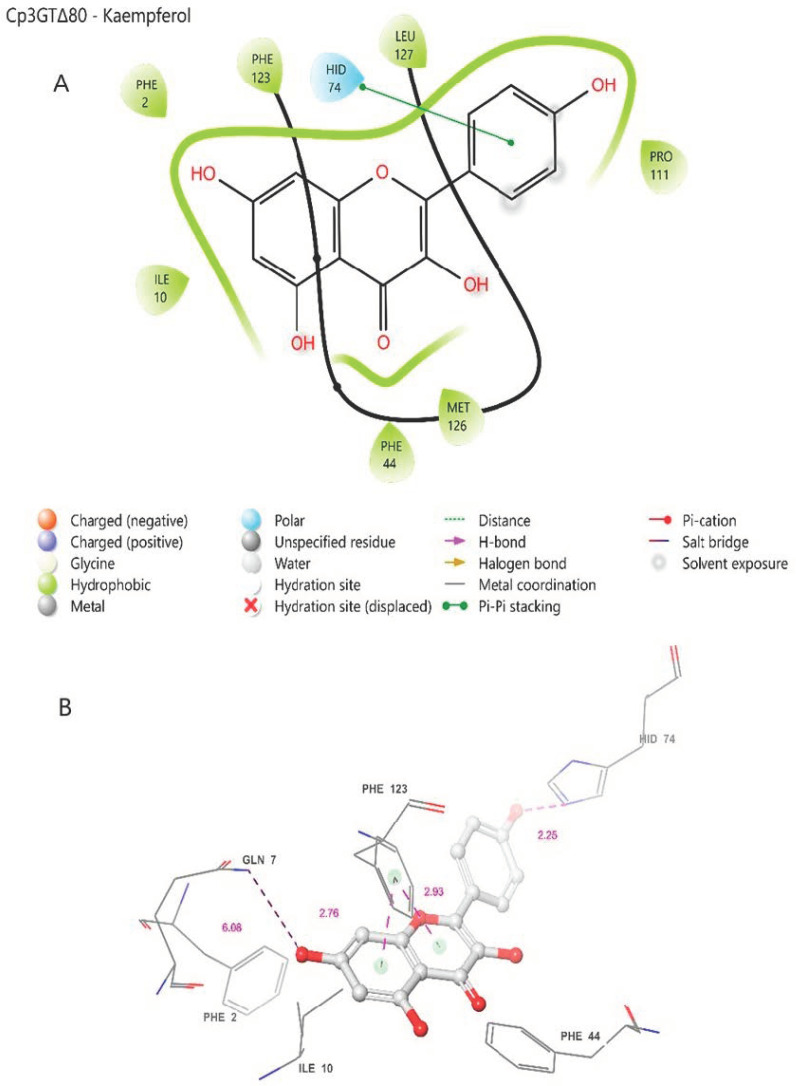
Two-dimensional (**A**) and three-dimensional models (**B**) of Cp3GTΔ80 residue–substrate interaction with kaempferol. All residues shown are positionally equivalent to native Cp3GT minus the 80 removed residues.

In the Cp3GTΔ80–kaempferol binding model, several differences compared to Cp3GT were observed. The omission of the catalytic residue HIS-22 in Cp3GTΔ80 is notable (Figure 8). Considering this evidence in the context of previous studies, it is strongly suggested that the absence of HIS-22 would render Cp3GTΔ80 inactive. The absence of critical docking residues, SER-20, PHE-17, and PHE-19, further supports this argument (Figure 8).

However, the model indicates the possibility that kaempferol can still bind to Cp3GTΔ80. Phenylalanine residues are predicted to be close enough to the A and C rings of kaempferol to establish pi bonds (Figure 8). Moreover, HID-74, which is equivalent to HID-154 in Cp3GT, may form a pi bond with the B ring (Figure 8A or a hydrogen bond with the B ring hydroxyl (Figure 8B. Coupled with the prediction that GLN-7 forms a hydrogen bond with the 7-OH of kaempferol and is analogous to GLN-87 in Cp3GT, this suggests that Cp3GTΔ80 might accommodate kaempferol binding. Yet, due to the absence of key residues, enzymatic glucosylation is improbable. While the Cp3GTΔ80 model shows potential for substrate binding, critical structural alterations hinder its enzymatic functionality. Direct experimental validation is needed to corroborate these in silico predictions.

### 3.11. Establishing the Flavonol Glucosylation Mechanism from VvGT1 as a Comparative Template for Cp3GT Variants

The COFACTOR-generated ligand binding sites of Cp3GT, Cp3GTΔ10 (see below), and Cp3GTΔ80 in interaction with the flavonols quercetin or kaempferol were extensively examined, and the specifics of their residue–ligand interactions were analyzed. One key observation was COFACTOR’s consistent selection of VvGT1 as the benchmark template for ligand binding with quercetin and kaempferol. Interestingly, flavonols are not the preferred substrate for VvGT1, having previously been shown to preferentially glucosylate the anthocyanidin cyanidin with 100 times higher efficacy than with quercetin [34,60]. However, co-crystallization was only achieved with quercetin and kaempferol, due to inherent challenges in stabilizing anthocyanidins during the crystallization process. This obstacle was later overcome when UGT78K6, an anthocyanidin 3-O GT with 43% sequence identity with Cp3GT, was co-crystallized with petunidin [36]. This divergence between the substrate preferences of VvGT1, UGT78K6, and Cp3GT is of significant interest. Specifically, VvGT1 can accommodate both anthocyanidins and flavonols, UGT78K6 glucosylates anthocyanidins with only trace activity with flavonols, while Cp3GT exclusively glucosylates flavonols [30,31,32,33,35,36,49].

Given the availability of crystal structures for VvGT1 co-crystallized with quercetin and kaempferol, combined with VvGT1′s 59% identity with Cp3GT (the highest among crystallized GTs), it is logical that VvGT1 with flavonol substrates would serve as the primary template for structural and ligand binding site predictions. As such, understanding the intricacies of VvGT1’s interactions with flavonols is paramount to understanding Cp3GT flavonol specificity. The following description is based on crystallographic analysis of VvGT1 crystallized with both quercetin and kaempferol by Offen et al. 2005 [34]. The mechanistic analysis is the same for both flavonols except where noted.

**Figure 9:**
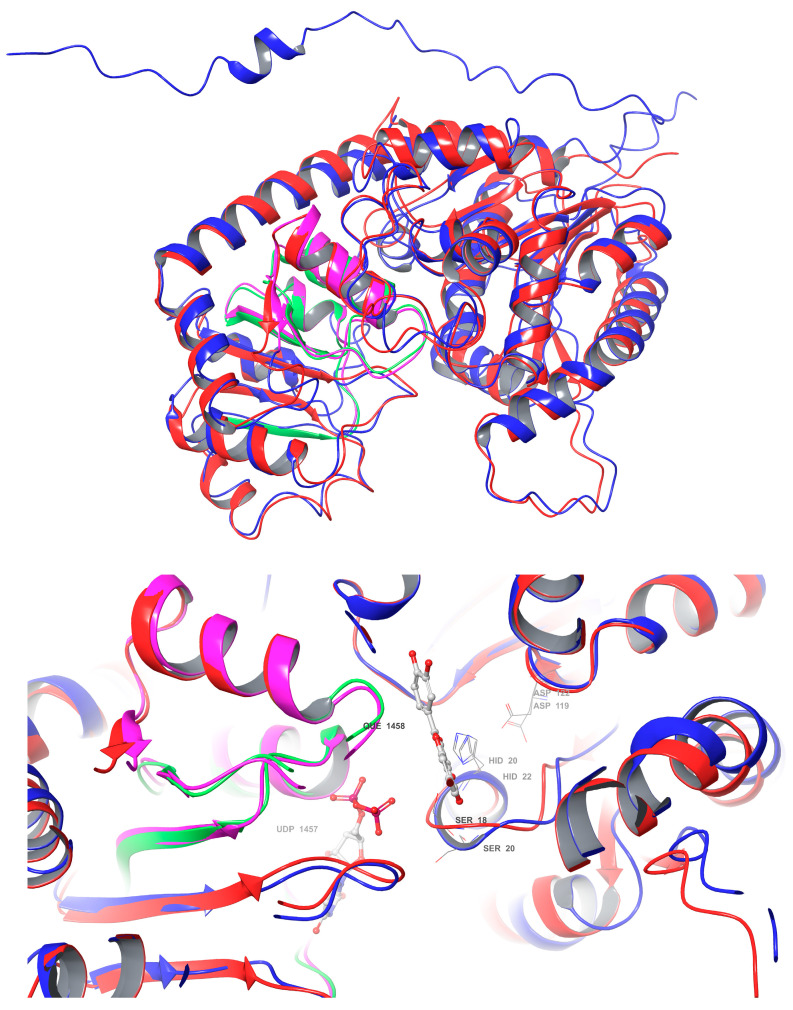
Structural superimposition of Cp3GT predicted model and VvGT1 crystal structure. (**Top**): Cp3GT (blue) and VvGT1 (red) showing the PSPG box in green for VvGT1 and pink for Cp3GT. (**Bottom**): Active site of Cp3GT predicted model and VvGT1 crystal structure showing UDP-glucose bound to residues in the PSPG box and quercetin bound to residues near the catalytic residues histidine, serine, and aspartic acid.

A comparative analysis of the structural features of Cp3GT and VvGT1 is shown in Figure 9. Both GTs have a similar 3D shape characteristic of GT-B enzymes, in which two β/α/β Rossmann-like domains face each other. The PSPG box, which defines the nucleotide donor binding site, is closely aligned in both enzymes (Figure 9). Furthermore, the acceptor binding site, including the catalytic residues histidine, serine, and aspartic acid, is also closely aligned (Figure 9), indicating a similar mechanism of binding and catalysis for both GTs. However, these models do not explain why VvGT1 can glucosylate flavonols and anthocyanidins, while Cp3GT can only glucosylate flavonols.

In the analysis of histidine residues within the protein structure, the tautomeric nature of the imidazole ring in histidine plays a crucial role. Imidazole can exist in two tautomeric forms, with the hydrogen atom shifting between the two nitrogen atoms. The HID form has the hydrogen attached to the δ-nitrogen (Nδ), which is bonded to the carbon in the double bond of the imidazole ring, while in the HIE form, the hydrogen is on the ε-nitrogen (Nε), which is not involved in the double bond.

This tautomerism adds a layer of complexity to the potential hydrogen bonding interactions with flavonol hydroxyl groups. It was initially assumed that the nitrogen atom closest to the hydroxyl group would be the primary participant in hydrogen bonding; under physiological conditions, either nitrogen atom could potentially be involved, depending on the tautomeric state of the histidine residue.

In the VvGT1 model, the placement of the double bond on the HID-20 imidazole ring does not clearly demonstrate the tautomeric nature of the imidazole ring, a limitation not addressed by existing software tools. However, considering the tautomeric flexibility of histidine, which can exist in two forms, with the hydrogen atom shifting between the two nitrogen atoms in the imidazole ring, it is plausible that both tautomeric forms could be involved in interactions with flavonols under physiological conditions. This tautomeric flexibility is particularly relevant in the active site of Cp3GT, influencing the ability of histidine residues to participate in hydrogen bonding and proton transfer.

In the case of Cp3GT, the tautomeric state of histidine residues, especially those involved in catalysis, may affect their interaction with flavonol substrates. This could determine the orientation and strength of hydrogen bonds formed with the substrate, thereby influencing the enzyme’s catalytic efficiency and specificity. Although current models provide a static representation, the dynamic tautomeric nature of histidine in a physiological environment could lead to variations in substrate binding and catalysis. Recognizing this, for clarity in Cp3GT binding predictions, it was assumed that the nitrogen atom closest to the hydroxyl group was involved in catalysis, irrespective of its labeling as HIS or HID, while acknowledging the inherent uncertainty due to the tautomeric nature of imidazole.

**Figure 10:**
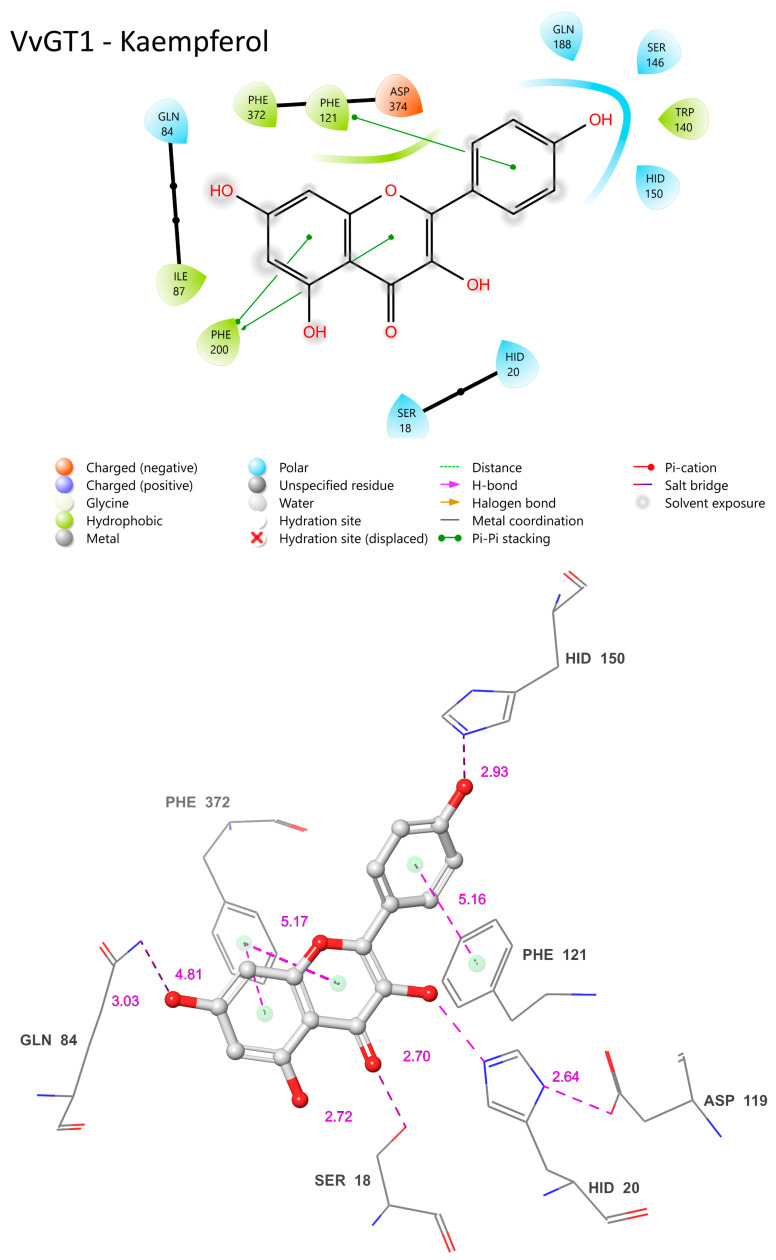
Visual representation of VvGT1 residue–substrate interaction with kaempferol. Two-dimensional (**top**) and three-dimensional (**bottom**) schematic of VvGT1 docked with kaempferol derived from crystal structure (PDB ID: 2c1Z). Distances shown as dashed lines with Å in pink.

The VvGT1 crystal structure indicates that the N-terminal HID-20 deprotonates the kaempferol 3-hydroxyl, priming it for nucleophilic attack by the glucose moiety (Figure 10) [34]. The protonation of HID-20 is stabilized by ASP 119. Concurrently, SER-18 forms a hydrogen bond with the C-ring carbonyl. Complementing this, HID-150 bonds with the B ring hydroxyl and GLN-84 with the 7-OH group; pi bonding interactions occur between the PHE-121/372 rings and the B/A ring, respectively (Figure 10). The torsional freedom of the B ring plays an important role in substrate orientation in the binding pocket and is oriented at an angle of 34.3°. This intricate network establishes the optimal orientation for glucosylation, positioning the catalytic HID-22 approximately 3.83 Å from the 3-OH catalytic site; this is a crucial arrangement for deprotonation (Figure 10). When quercetin is the acceptor substrate, the mechanism differs only in that the 3′ hydroxyl on the B ring (not present in kaempferol) interacts with the main chain carbonyl of a GLU-189 through a water-mediated hydrogen bond. Additionally, the quercetin B ring is oriented at a 25.2° angle.

### 3.12. Comparative Analysis of Cp3GT and VvGT1 Ligand Binding Dynamics: Insights and Implications

**Figure 11:**
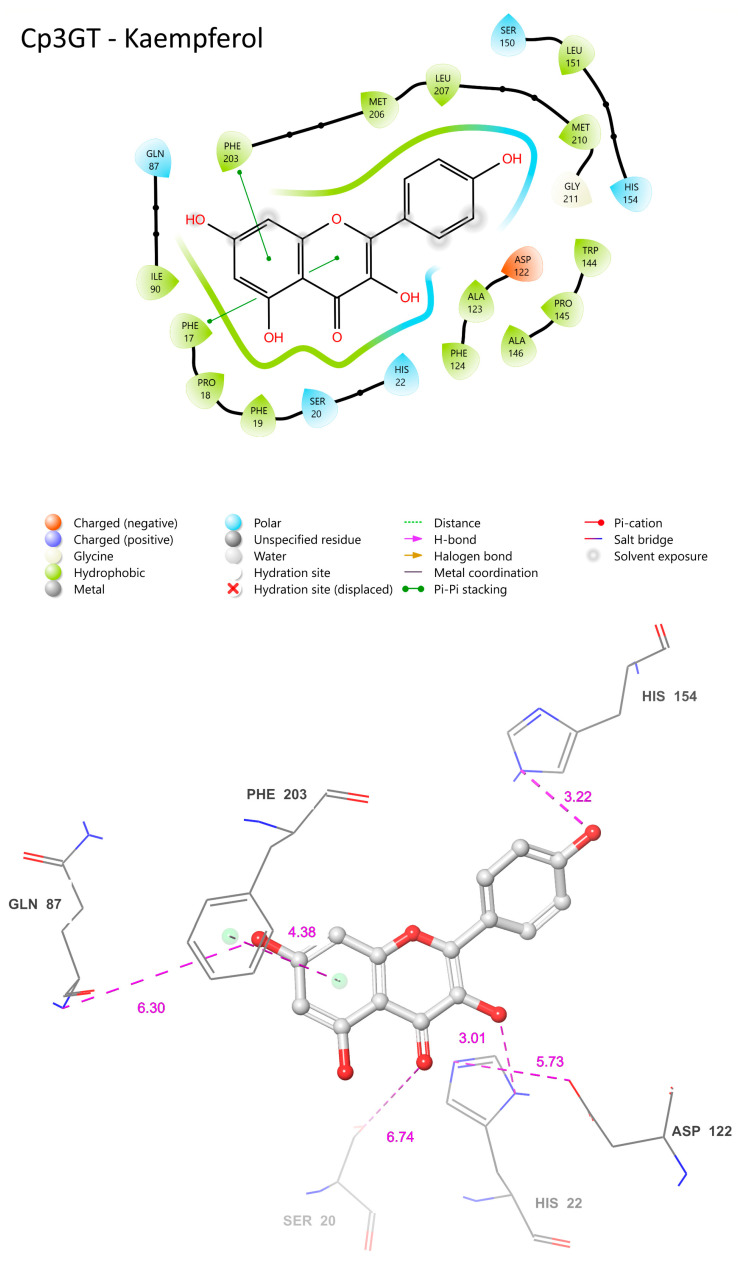
Visual representation of Cp3GT residue–substrate interaction with kaempferol. Two-dimensional (**top**) and three-dimensional (**bottom**) schematic of Cp3GT bound with kaempferol derived from COFACTOR.

The COFACTOR-predicted ligand binding sites for Cp3GT docked with kaempferol are consistent with the VvGT1 mechanism. In Cp3GT, HID-22 is positioned 3.01 Å away from the 3-OH catalytic site (Figure 11), a slightly greater distance than the 2.70 Å observed for the analogous residue in VvGT1 (Figure 10). Considering histidine’s conserved catalytic function across various GTs and given its inherent flexibility, it is highly likely that even if the model indicates a greater distance, HID-22 retains a similar functional role as that observed in VvGT1.

Although ASP-119 is anticipated to stabilize HID-22, facilitating its deprotonation of the 3-OH site, it is positioned 5.73 Å away (Figure 11), in contrast with the closer 2.64 Å distance seen between VvGT1’s ASP-119 and HID-20 (Figure 10). While this difference might be due to the limitations or specificities of the modeling approach, it is important to consider that ASP-119 and HID-22 could still interact in a similar manner to that observed in VvGT1. Clarification of their actual spatial relationship and functional interactions in vivo will require further experimental validation.

SER-20 is a key residue for flavonol 3-O glucosylation in Cp3GT, as indicated by a loss of activity in a mutant where SER-20 was replaced by leucine [61]. However, in the current analysis, its predicted distance of 6.74 Å from the C ring carbonyl (Figure 11) stands in contrast to the 2.72 Å separating SER-18 and the same carbonyl in VvGT1 (Figure 10). This discrepancy implies a lack of hydrogen bond formation in Cp3GT, which contradicts previous docking models that positioned SER-20 much closer to the C ring carbonyl but, notably, did not propose a mechanism for SER-20’s interaction with the flavonol [33]. In VvGT1, SER-18, which is analogous to SER-20 in Cp3GT, plays a crucial role by hydrogen bonding to the C ring carbonyl of flavonol quercetin, stabilizing its position within the binding pocket. The divergence between these observations and the current predictions underlines the challenges and nuances inherent in different modeling approaches and emphasizes the need for experimental validation [33].

Positionally equivalent residues, GLN-87 and HIS-154, in Cp3GT are predicted to engage in hydrogen bonding with the 7-OH and B ring hydroxyl, respectively (Figure 11). These predictions align well with the interactions observed in VvGT1, underscoring possible evolutionarily conserved substrate binding strategies. However, the 6.38 Å distance between Cp3GT’s PHE-124 and the A ring (Figure 11) is notable. While this distance suggests that PHE-124 may not be directly involved in pi bonding as in VvGT1, one must consider that subtle differences in protein conformation could bring it closer during the catalytic cycle. Interestingly, although PHE-203 is not a direct positional counterpart in the sequences, its proximity of 4.38 Å from the A ring suggests a compensatory pi bonding mechanism like VvGT1. Similarly, PHE-17, being 4.42 Å from the C ring, is poised to form a pi bond, mirroring VvGT1’s PHE-372 (Figure 10). The observation that both Cp3GT and VvGT1 have an identical 34.3° torsional angle for the B ring suggests the importance of this alignment for substrate interaction.

### 3.13. Cp3GT’s Ligand Interactions with Quercetin and Kaempferol: Conserved Features and Functional Divergences

A ligand binding site prediction was conducted for Cp3GT with quercetin given that it is the preferred substrate for Cp3GT [31,32,33,49]. This was reinforced by COFACTOR’s selection of quercetin as the binding template for both Cp3GT and Cp3GTΔ10. However, for Cp3GTΔ80, COFACTOR identified kaempferol as the binding template. To provide a comprehensive understanding, it was deemed essential to present models of Cp3GT interacting with both substrates. Although quercetin stands as the preferred substrate for Cp3GT, the enzyme readily glucosylates kaempferol [30,31,32,33,49].

**Figure 12:**
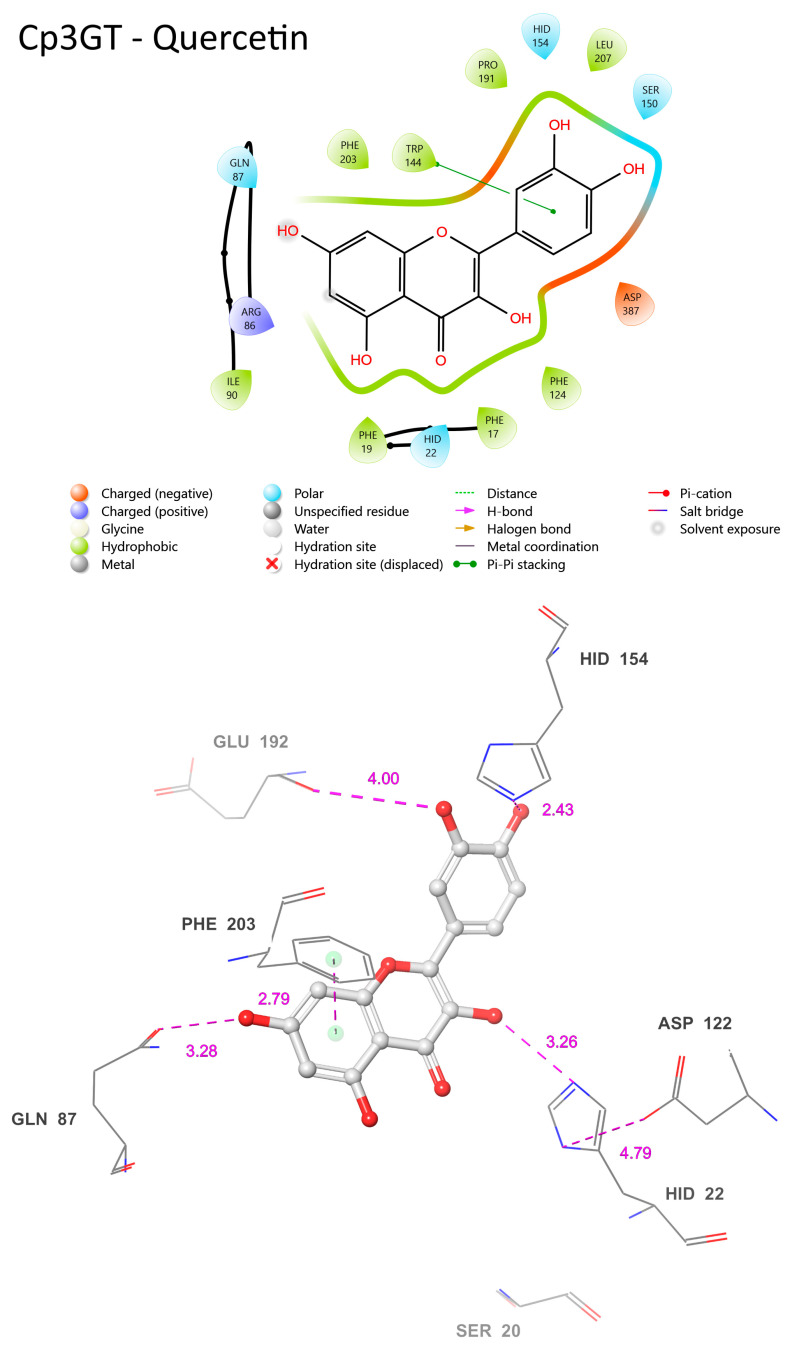
Visual representation of Cp3GT residue–substrate interaction with quercetin. Two-dimensional (**top**) and three-dimensional (**bottom**) schematic of Cp3GT docked with quercetin derived from COFACTOR.

The common features of Cp3GT when bound to quercetin or kaempferol encompass residues such as HID-22, GLN-87, ASP-122, HID-154, and PHE-203 (Figure 11). These residues maintain similar positions and proximity to the substrate across both interactions. Their conservation across different ligand binding scenarios suggests a vital role in the enzyme’s substrate binding or catalytic activities. For instance, HID-22’s 3.26 Å distance from the 3-OH bound to quercetin (Figure 12) compared with 3.01 Å distance from that bound to kaempferol (Figure 11) reinforces the role of histidine as the catalytic Brønsted base, and supports the biochemical data showing Cp3GT’s ability to glucosylate both flavonols [30,31,32,33,49]. It further highlights the conserved nature of this residue’s role across diverse glycosyltransferases.

In both models, ASP-122’s role in stabilizing HID-22 is evident. The distance between these residues is 4.79 Å when Cp3GT is bound to quercetin (Figure 12) and 5.73 Å with kaempferol (Figure 11). Given the known function of aspartic acid in supporting the charge of histidine during GT catalysis [16,34,36], it is anticipated that this interaction remains functionally consistent, irrespective of whether the substrate is kaempferol or quercetin. Furthermore, the distance between ASP-122 and HID-22 could exhibit variability in vivo due to the torsional flexibility of the histidine imidazole ring and the dynamic nature of these residues.

**Figure 13:**
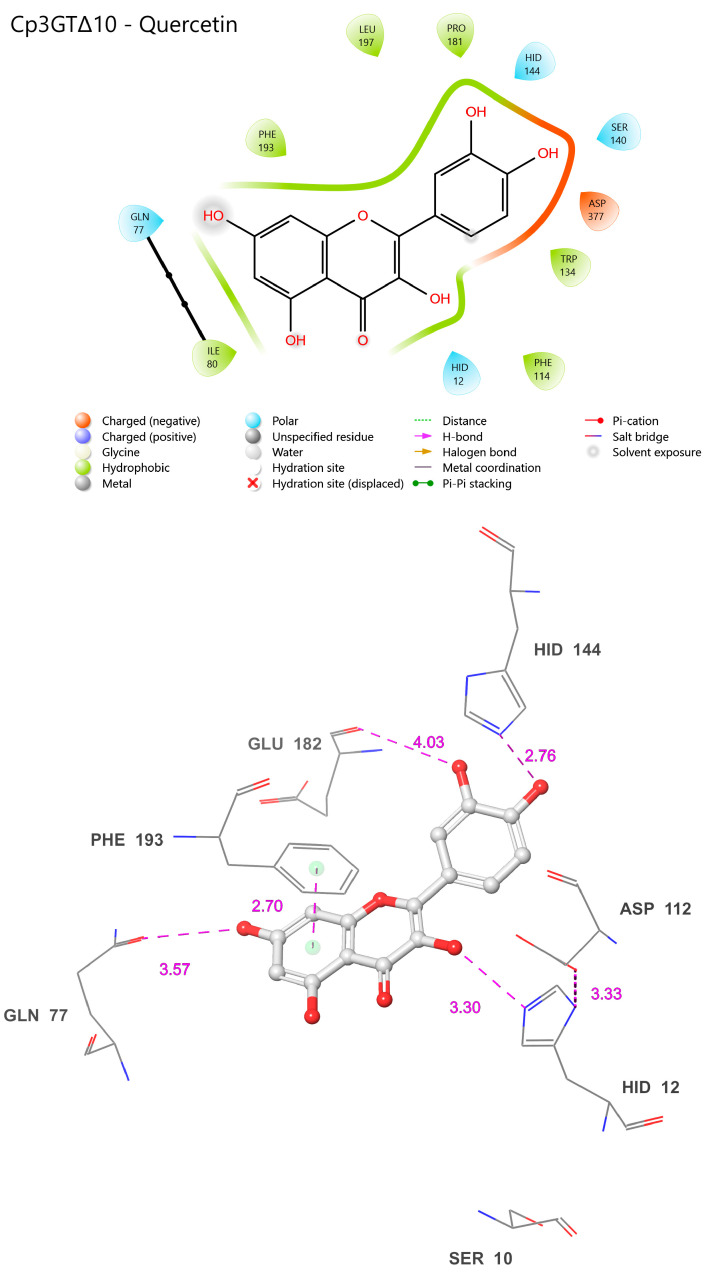
Visual representation of Cp3GTΔ10 residue–substrate interaction with quercetin. Two-dimensional (top) and three-dimensional (bottom) schematic of Cp3GTΔ10 docked with quercetin derived from COFACTOR. All residues shown are positionally equivalent to Cp3GT shown in Figure 12, minus the 10 removed residues.

There are distinct variations as well. The prediction of phenylalanine rings pi bonding with flavonol rings highlights the significance of this interaction in substrate binding (Figure 11). However, the PHE-17 pi bond with the C ring was not predicted to occur in Figure 12. This further contrasts with phenylalanine pi-bonding with flavonol rings shown in Figure 13; see also [36]. The absence or alteration of these interactions could affect substrate binding and may play a role in substrate specificity. A disrupted or weakened pi bond interaction could potentially compromise the overall docking stability, suggesting that, while subtle, these interactions might play crucial roles in the enzyme’s catalytic efficiency.

SER-20′s location in the current model is even more remote than the already significant 6.74 Å distance seen when Cp3GT is bound to kaempferol (Figure 10). This increasingly distant positioning makes a direct interaction between SER-20 and the C ring carbonyl highly improbable (Figure 11). This raises interesting questions about the flexibility and movement of the enzyme during the catalysis process. Given the evidence from biochemical studies showing a drastic loss of Cp3GT activity when SER-20 is mutated to leucine [61], the functional implications of such positional discrepancies between the modeled and actual states is of great interest. The contradiction between the current observation and previous docking models, which posited SER-20 in a much closer proximity to the flavonol [33], underlines the inherent challenges of in silico predictions. It serves as a pertinent reminder of the need to correlate computational models with empirical evidence and, where discrepancies arise, to further refine modeling approaches.

A potential pi bond interaction between TRP-144 and the B ring is observed (Figure 11). The presence of such an interaction brings to light the versatile nature of tryptophan residues in protein–ligand interactions. Tryptophan, with its unique indole ring, often plays pivotal roles in protein function, be it in ligand binding or in facilitating protein conformational changes. The observed interaction with TRP-144 might provide additional stabilization to the flavonoid within the active site, potentially ensuring efficient catalysis.

### 3.14. Structural Conservation and Divergences in Cp3GTΔ10: Implications for Binding Affinity and Enzymatic Activity

The models of Cp3GTΔ10 exhibited minimal deviation from Cp3GT in terms of catalytic and docking residues (Figure 13). This observation suggests that the deletion in Cp3GTΔ10 might not significantly alter the core structure of the protein, preserving its substrate binding pocket intact. This structural conservation, even after deletions or mutations, emphasizes the evolutionary resilience of enzymatic architectures, designed to maintain functionality over various alterations.

However, a notable divergence was observed concerning the aromatic residues PHE-17 and PHE-19. In the Cp3GT model, both residues were posited to be proximal to the A and C rings, with PHE-17 even speculated to form a pi bond with the C ring in the kaempferol-bound state (Figure 11). Conversely, in Cp3GTΔ10, neither PHE-17 nor PHE-19 are sufficiently close to establish such interactions with the A and C rings (Figure 13). This potential lack of pi–pi interactions in Cp3GTΔ10 could influence binding affinity and GT activity. While individual pi–pi interactions may not be particularly strong, cumulatively they play a pivotal role in stabilizing protein–ligand complexes.

While the in silico models provide valuable insights into the structural and functional dynamics of Cp3GT and its variants, it is crucial to acknowledge their limitations. Notably, the predicted positioning of SER-20 in the Cp3GT models conflicts with empirical data from mutational studies. These studies have shown that the alteration of SER-20 significantly impacts the enzymatic activity of Cp3GT [61], suggesting a closer proximity to the flavonol substrate than predicted by the models. This discrepancy highlights a limitation of the current modeling approaches, particularly in capturing the dynamic flexibilities of enzyme structures during the catalytic process. The inherent flexibility of proteins, influenced by their interaction with substrates and the cellular environment, may not be fully represented in static models. Therefore, while these models offer valuable structural predictions, they should be interpreted with caution and supplemented with empirical data for a more comprehensive understanding of enzyme function.

Interestingly, the experimental data showed reduced GT activity for Cp3GTΔ10 (Figure 3). Altered folding within the catalytic cleft could attenuate the enzyme’s activity by impacting substrate binding and/or catalytic efficiency. It is also plausible that the carryover between peaks during the MonoQ chromatographic purification step resulted in sample heterogeneity, complicating the accurate assessment of its enzymatic efficiency. Regardless of this, the reduced activity in conjunction with the observed structural differences raises pertinent questions about Cp3GTΔ10’s enzymatic behavior.

To empirically validate the association between loss of residues due to truncation and the consequent loss of enzymatic activity in Cp3GT, as predicted by the in silico models, future research endeavors could focus on comprehensive enzyme kinetics studies to decipher the mechanistic underpinnings of Cp3GTΔ10′s altered activity. This may involve employing steady-state kinetics to precisely determine key enzymatic parameters such as the Michaelis constant (K_m_), maximum reaction velocity (V_max_), and catalytic efficiency, which will elucidate the enzyme’s affinity for its substrate and its maximal catalytic capability under defined conditions. Furthermore, assessing initial velocity (V_0_) under various substrate concentrations will provide insights into the enzyme’s activity and potential inhibition under initial reaction conditions, thereby providing a comprehensive understanding of the catalytic discrepancies observed in Cp3GTΔ10.

This can be achieved by constructing expression vectors with sequential truncations of the Cp3GT sequence, mirroring the deletions analyzed in the computational models. The expressed and purified truncated proteins would then be tested for glycosyltransferase activity against flavonols like quercetin and kaempferol. Through this experimental approach, the activity levels of these truncated enzymes could be quantitatively compared with the full-length Cp3GT and computational predictions, thereby experimentally validating the impact of truncation on enzymatic activity. This method will enable a direct correlation of structural changes with functional outcomes, significantly enhancing the understanding of Cp3GT’s structure–function dynamics.

## 4. Conclusions

In this study, key challenges encountered in the expression of Cp3GT in *Pichia pastoris*, a common system for recombinant protein production, were addressed. A significant issue was proteolytic degradation by native Pichia proteases, which was mitigated by modifying cell handling and storage procedures. Immediate lysis post-induction and prompt treatment with PMSF and glycerol effectively reduced Cp3GT degradation, enhancing protein integrity, as was evident in subsequent purification steps.

Another challenge was the optimization of the yield and purity during production scale-up. The high cell density in large-scale cultures often led to inefficient lysis and non-specific binding in chromatography. This was addressed by adjusting the lysis method and fine-tuning the chromatography protocol; notably, a shallower gradient in anion exchange chromatography was used, and size exclusion chromatography was incorporated as a final step. These changes significantly improved the separation of Cp3GT from the contaminants, as shown by the distinct elution patterns and higher purity of the final product.

These experiences highlight the need for strategic adaptations in *Pichia pastoris* protein expression, particularly in managing protease activity and optimizing chromatography for scale-up. The modifications implemented contribute valuable insights into the enhancement of the efficiency and reliability of recombinant protein production in yeast systems. In this study, a detailed in silico exploration of Cp3GT’s ligand binding interactions with the flavonols quercetin and kaempferol was conducted, and this analysis was extended to its truncated versions, Cp3GTΔ10 and Cp3GTΔ80. These evaluations were set against the backdrop of VvGT1, an anthocyanidin/flavonol GT with a resolved crystal structure and a well-established mechanism for flavonol glucosylation. This comparison provides a broader context with which to understand the functional catalytic and docking residues of Cp3GT, as elucidated by the existing biochemical data.

The integration of modern structural modeling tools, specifically D-I-TASSER, with rapid ligand binding predictors like COFACTOR, presents a significant development in the probing of enzyme–ligand interactions. Although COFACTOR does not utilize rigorous docking algorithms, its predictions are notably consistent with data from crystal structures and previous Cp3GT binding analyses using AutoDock. This synergy between D-I-TASSER and COFACTOR reveals the operational nuances of Cp3GT, despite some minor discrepancies observed.

The current models, while advanced, may not accurately represent the dynamic nature of protein–ligand interactions. Specifically, the positioning of SER-20 in the models is inconsistent with the empirical mutational data, which suggests a more integral role of this residue in the enzyme’s catalytic activity. This incongruity underscores the need for refinement in the modeling techniques, particularly in terms of incorporating protein flexibility and dynamic conformational changes. Future modeling efforts could benefit from the integration of data from molecular dynamics simulations or by employing more advanced algorithms that better capture the dynamic nature of protein structures. This would enhance the predictive accuracy of the models, making them more aligned with experimental observations.

However, a major challenge is the lack of a resolved crystal structure for a GT with intrinsic flavonol specificity, which limits a comprehensive understanding of Cp3GT’s selective glucosylation mechanism at the 3-OH position. The meticulous expression and purification of Cp3GT, as demonstrated in this research, sets a foundation for future structural evaluations, potentially through X-ray crystallography. Such future efforts in achieving a crystal structure of Cp3GT, particularly when co-crystallized with a flavonol substrate, are anticipated to provide critical insights into the enzyme’s specificity.

In conclusion, this research has significantly advanced the understanding of the structural and functional aspects of Cp3GT, a key plant flavonol-3-O glucosyltransferase from *Citrus paradisi*. Through meticulous expression and purification processes, coupled with in-depth in silico structural analyses, the study has illuminated key insights into Cp3GT’s interaction with flavonols and the implications of its structural nuances. The use of modern computational tools like D-I-TASSER and COFACTOR has been instrumental in exploring the enzyme’s ligand binding dynamics and offering predictive insights into its substrate specificity.

The lack of a resolved crystal structure for Cp3GT presents an ongoing challenge; however, the groundwork laid by this study establishes a robust foundation for future structural analyses, possibly through X-ray crystallography. Such advances would not only enhance understanding of Cp3GT but also hold promise for wider applications in enzyme engineering and synthetic biology. The research underscores the evolving capabilities in computational modeling and its critical role in elucidating enzyme mechanisms, setting the stage for transformative discoveries in the field of biotechnology.

The findings of this study on Cp3GT, particularly the structural insights and enzymatic mechanisms, have far-reaching implications for synthetic biology and pharmaceutical applications. The ability to manipulate and engineer enzymes like Cp3GT opens avenues for the tailored synthesis of complex flavonoid glycosides, which are pivotal in developing novel therapeutic agents. Flavonoids, known for their antioxidant and anti-inflammatory properties, hold significant potential in pharmaceuticals, especially in targeting diseases where oxidative stress and inflammation play a critical role. By demonstrating the importance of specific residues in Cp3GT’s enzymatic activity and substrate specificity, this research provides a blueprint for the engineering of flavonol-specific glycosyltransferases with enhanced efficiency or altered substrate preference. Such engineered enzymes could be pivotal in synthesizing flavonol derivatives with improved solubility, stability, and bioavailability, making them more effective as pharmacological agents. Furthermore, the application of these findings in synthetic biology could lead to the development of novel biosynthetic pathways, enabling the cost-effective production of valuable flavonoids and their derivatives, and could thereby have a profound impact on the fields of drug discovery and biotechnological innovation.

## Figures and Tables

**Table 1:** COFACTOR scoring parameters for Cp3GT (modeled with recombinant tags) and two N-terminal truncated versions as compared to VvGT1.

Name	PDB Analog	C-Score	TM-Score	RMSD	IDEN	Coverage	BS-Score	Ligand
Cp3GT	2c9zA (VvGT1)	0.34	0.842	1.41	0.596	0.869	1.75	Quercetin
Cp3GTΔ10	2c9zA (VvGT1)	0.34	0.854	1.38	0.599	0.878	1.72	Quercetin
Cp3GTΔ80	2c1zA (VvGT1)	0.33	0.836	1.45	0.598	0.863	1.47	Kaempferol

## Data Availability

The original contributions presented in the study are included in the article/supplementary material. Additional data presented in this study are available in East Tennessee State University Digital Commons at https://dc.etsu.edu/etd/4321/, accessed on 10 November 2023.

## Supplementary Materials

The following supporting information can be downloaded at: https://www.mdpi.com/article/10.3390/biotech13010004/s1, Figure S1: Linear Schematic of Cp3GT Protein Construct. Supplementary Figure S2. Growth and Expression Analysis of *P. pastoris* Transformed with Recombinant Cp3GT. Supplementary Figure S3. Cobalt-affinity Chromatography Purification Profile. Supplementary Figure S4. MonoQ Anion Exchange Chromatography of Cobalt-Affinity Purified Cp3GT Eluate #1. Supplementary Figure S5. MonoQ Anion Exchange Chromatography of Cobalt-Affinity Purified Cp3GT Eluate #2. Supplementary Figure S6. Cp3GT Cobalt-affinity and MonoQ Purification Profile #2. Supplementary Figure S7. Cp3GT Cobalt-affinity and MonoQ Anion Exchange Purification Profile #3. Supplementary Figure S8. Cp3GT Cobalt-affinity and MonoQ Anion Exchange Purification Profile #4. Table S1: COFACTOR Scoring Parameters for Cp3GT (Modeled Without Recombinant Tags) and Two N-Terminal Truncated Versions as Compared to VvGT1. Figure S9: 2D And 3D Models of Cp3GTΔ80 (Modeled Without Tags) Residue-Substrate Interaction with Quercetin. Figure S10: Visual Representation of Cp3GT (Modeled Without Tags) Residue-Substrate Interaction with Kaempferol. Figure S11: Visual Representation of Cp3GTΔ10 (Modeled Without Tags) Residue-Substrate Interaction with Quercetin.

## Abbreviations

Glycosyltransferase	GT
Flavonol specific 3-O glucosyltransferase	Cp3GT
Uridine diphosphate glycosyltransferase	UGT
Phenylmethylsulfonyl fluoride	PMSF
β-mercaptoethanol	BME
Alcohol oxidase	AOX
Optical density	OD
Yeast peptone dextrose	YPD
Buffered minimal glycerol yeast media	BMGY
Buffered minimal methanol yeast media	BMMY
Distance-Guided Protein Structure Prediction	DI-TASSER
Multiple sequence alignment	MSA
