# Supplementary material for: Expression and Purification of Cp3GT: Structural Analysis and Modeling of a Key Plant Flavonol-3-O Glucosyltransferase from Citrus paradisi
Source: BioTech (Basel). 2024 Feb 7;13(1):4. doi: 10.3390/biotech13010004 (PMC10885057; doi:10.3390/biotech13010004)
Supplement: Supplementary file 1 [file biotech-13-00004-s001.zip › biotech-2758577-supplementary.pdf]

Supplementary Materials

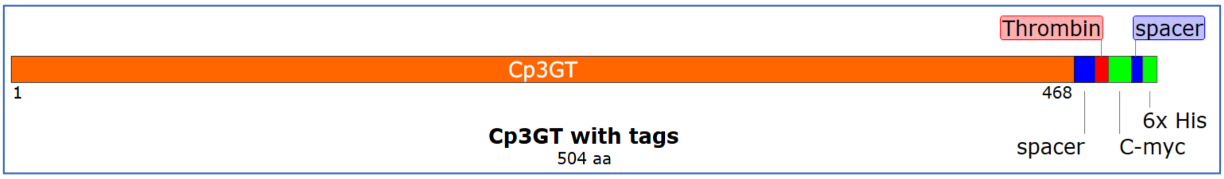

Figure S1: Linear Schematic of Cp3GT Protein Construct. The 468 amino acid sequence of Cp3GT is shown in orange. Thrombin cleavage sequence is shown in red downstream of Cp3GT and separated by a small spacer sequence shown in blue. C-myc and 6x His tag are shown in green separated by another small spacer sequence shown in blue.

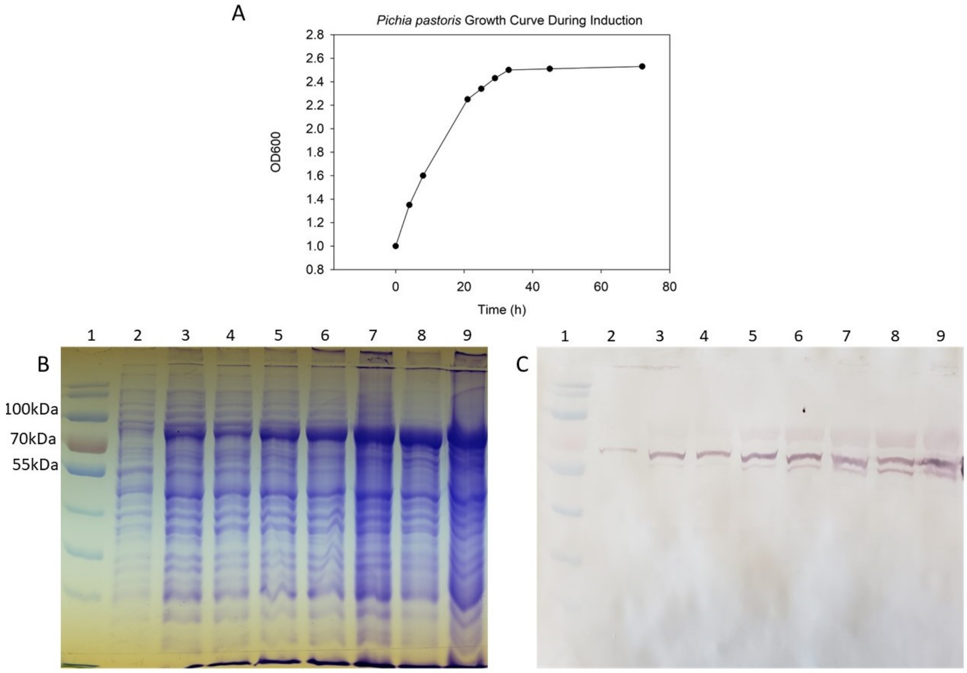

Figure S2. Growth and Expression Analysis of *P. pastoris* Transformed with Recombinant Cp3GT. A. Growth curve during induction. B. Cp3GT Expression Profile, Coomassie stained, and C. immunoblot loaded with 1) Molecular Weight Marker (MWM), 2) 4 h, 3) 8 h, 4) 21 h, 5) 25 h, 6) 29 h, 7) 33 h, 8) 45 h, 9) 72 h.

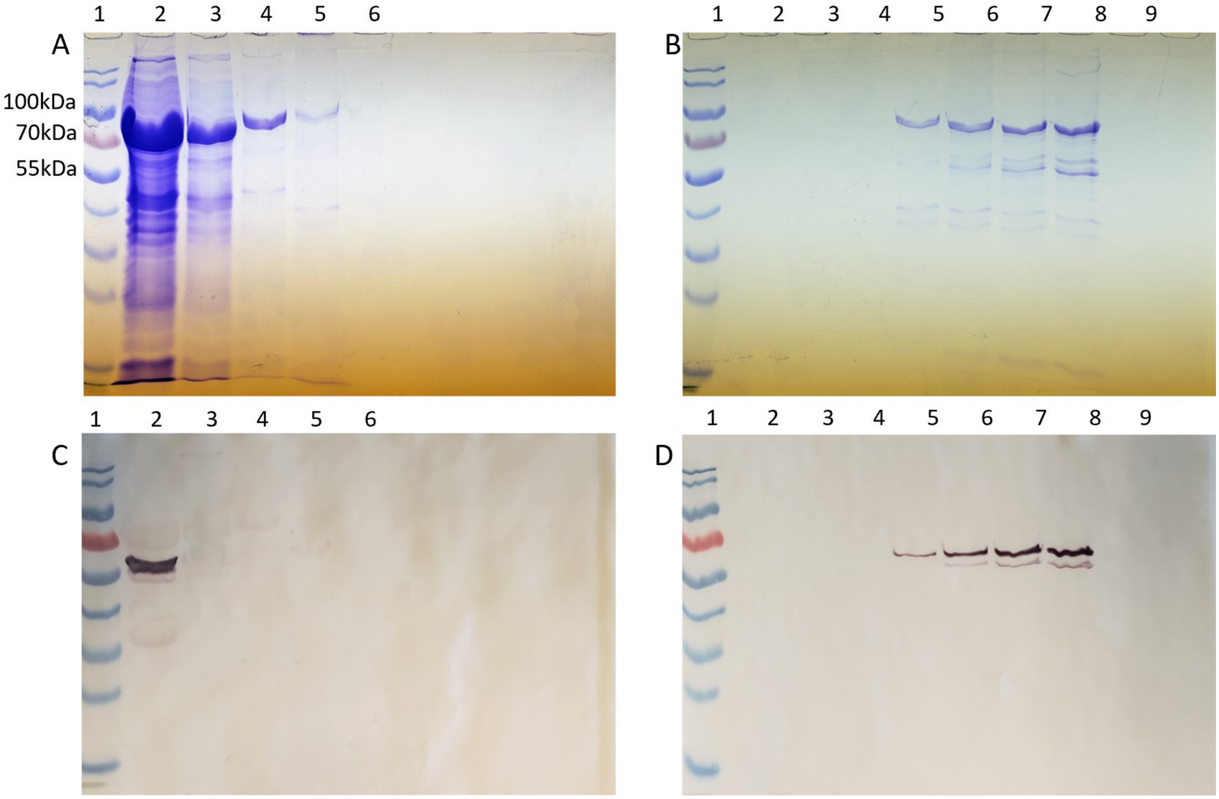

Supplementary Figure S3. Cobalt-affinity Chromatography Purification Profile. A. Coomassie stain and C. immunoblot loaded with 1. MWM, 2. Crude lysate, 3. Flowthrough, 4. Wash 1, 5. Wash 2, 6. Wash 3. B. Coomassie stain and D. immunoblot loaded with 1. MWM 2. Elution 1, 3. Elution 2, 4. Elution 3, 5. Elution 4, 6. Elution 5, 7. Elution 6, 8. Elution 7, 9. Elution 8. Wash fractions were collected every 50 mL. Elution fractions were collected every 0.5 mL.

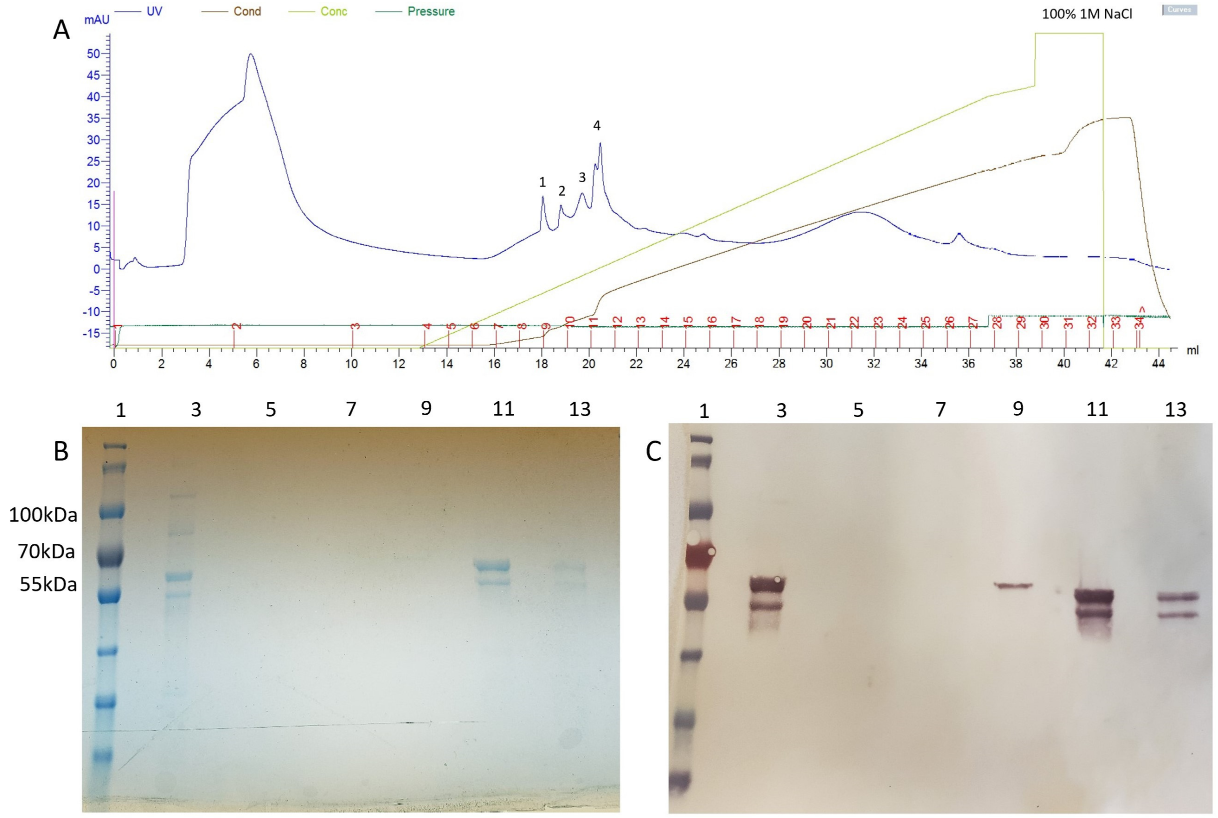

Supplementary Figure S4. MonoQ Anion Exchange Chromatography of Cobalt-Affinity Purified Cp3GT Eluate #1. A. MonoQ chromatogram showing 4 peaks of interest labeled 1-4 eluted between 130-250 mM NaCl, eluted using a 0-100% gradient of Buffer B over 30 mL collecting 1 mL fractions. The y-axis is milli-absorbance units (mAU) and the x axis is mL buffer (black). Fraction numbers are denoted as red dashes along the x-axis. B. Coomassie stain and C. immunoblot loaded with 1. MWM, 3. Pooled cobalt affinity eluate, 5. Peak 1, 7. Peak 2, 9. Peak 3, 11. Peak 4 (Fraction 11), 13. Peak 4 (Fraction 12). All other lanes contained loading dye.

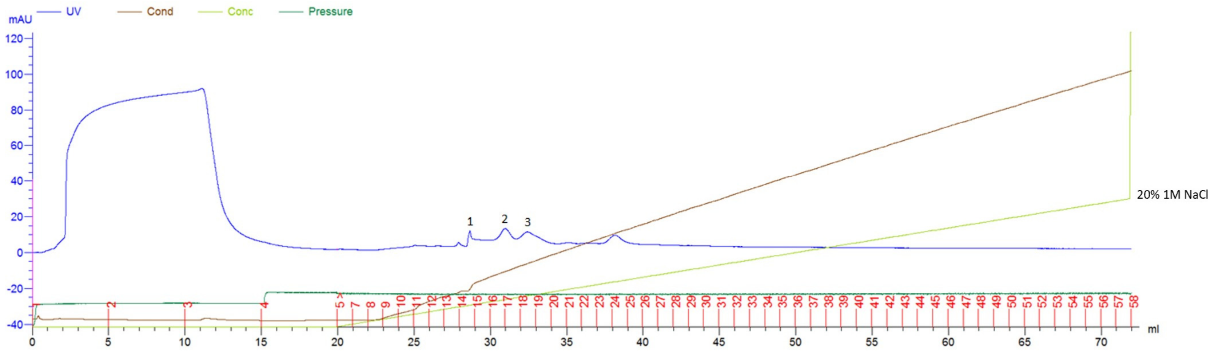

Supplementary Figure S5. MonoQ Anion Exchange Chromatography of Cobalt-Affinity Purified Cp3GT Eluate #2. The y-axis is milli-absorbance units (mAU) and the x axis is mL buffer (black). Fraction numbers are denoted as red dashes along the x-axis. Three peaks of interest labeled 1-3

eluted at 72, 92, and 104mM NaCl, respectively, using a 0-20% gradient of Buffer B over 50 mL. Fractions were collected in 0.5 mL increments.

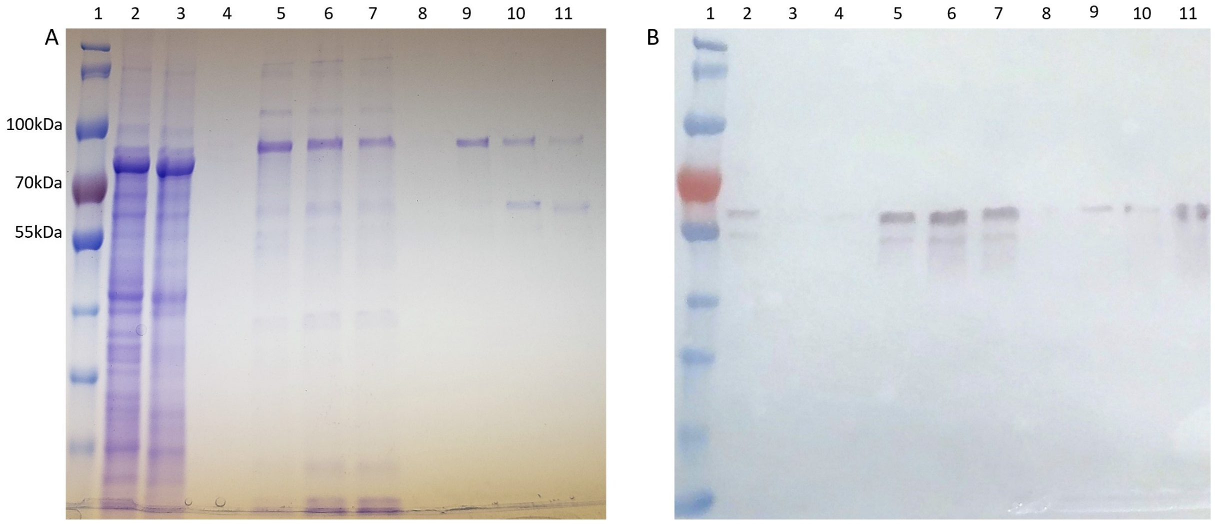

Supplementary Figure S6. Cp3GT Cobalt-affinity and MonoQ Purification Profile #2. A. Coomassie stain and B. immunoblot loaded with 1. MWM, 2. Crude lysate, 3. Cobalt-affinity flowthrough, 4. Affinity elution fraction 1, 5. Affinity elution fraction 2, 6. Affinity elution fraction 3, 7. Affinity elution fraction 4, 8. Loading dye, 9. MonoQ peak 1, 10. MonoQ peak 2, 11. MonoQ peak 3. Affinity elution fractions were collected every 1mL.

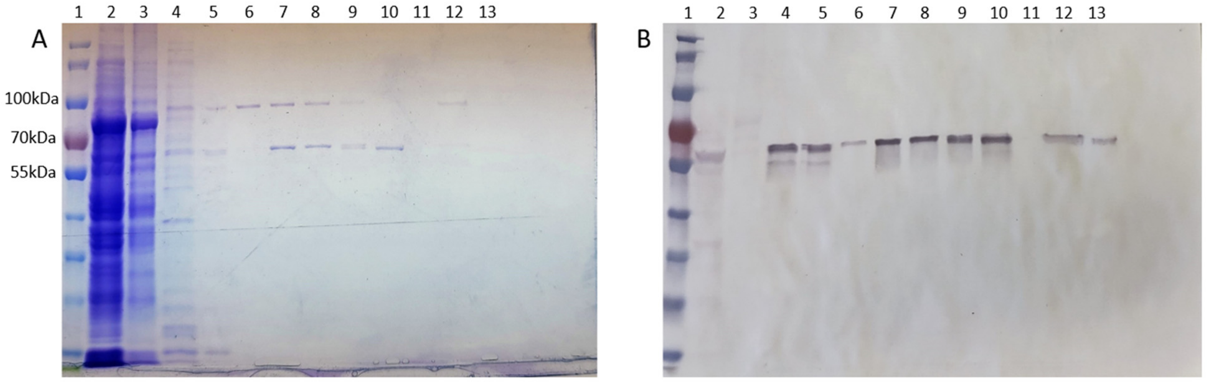

Supplementary Figure S7. Cp3GT Cobalt-affinity and MonoQ Anion Exchange Purification Profile #3: A. Coomassie stain and B. immunoblot loaded with 1. MWM, 2. Crude lysate, 3. Cobalt-affinity flowthrough, 4. Cobalt-affinity eluate pooled 1, 5. Cobalt-affinity eluate pooled 2, 6. Peak A (fraction 17), 7. Peak A (fraction 18), 8. Fraction 19, 9. Peak B (fraction 20), 10. Peak B (fraction 21), 11. Loading dye, 12. Cp3GT control, 13. Cp3GT control.

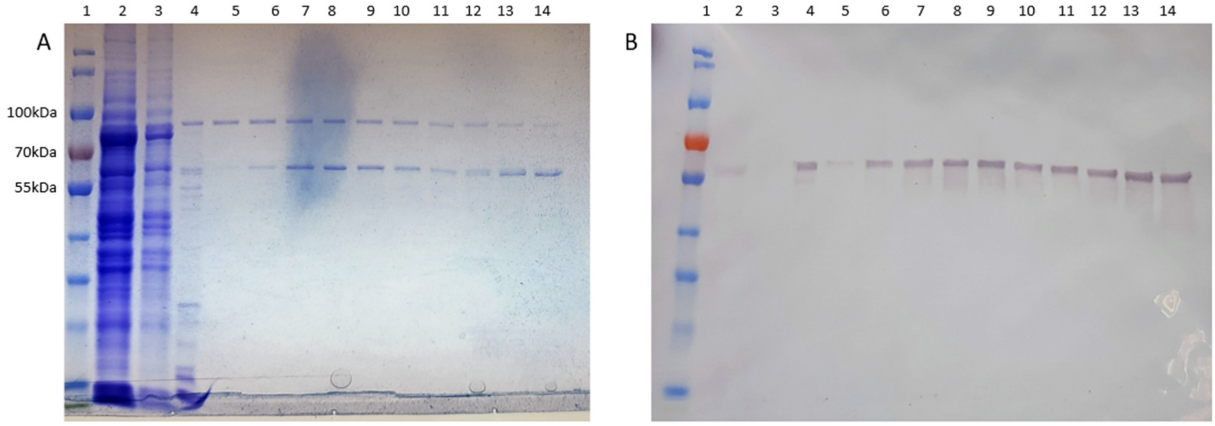

Supplementary Figure S8. Cp3GT Cobalt-affinity and MonoQ Anion Exchange Purification Profile #4. A. Coomassie stain and B. immunoblot loaded with 1. MWM, 2. Crude lysate, 3. Affinity flowthrough, 4. Pooled affinity eluate, 5-10. Peak A fractions 35-40, 11-14. Peak B fractions 41-44.

Table S1: COFACTOR Scoring Parameters for Cp3GT (Modeled Without Recombinant Tags) and Two N-Terminal Truncated Versions as Compared to VvGT1

Name	PDB Analog	C Score	TM-Score	RMSD	IDEN	Coverage	BS-Score	Ligand
Cp3GT (no tags)	2c1zA (VvGT1)	0.35	0.907	1.36	0.597	0.934	1.72	Kaempferol
Cp3GTΔ10 (no tags)	2c9zA (VvGT1)	0.36	0.916	1.36	0.602	0.943	1.77	Quercetin
Cp3GTΔ80 (no tags)	2c9zA (VvGT1)	0.34	0.907	1.62	0.599	0.943	1.47	Quercetin

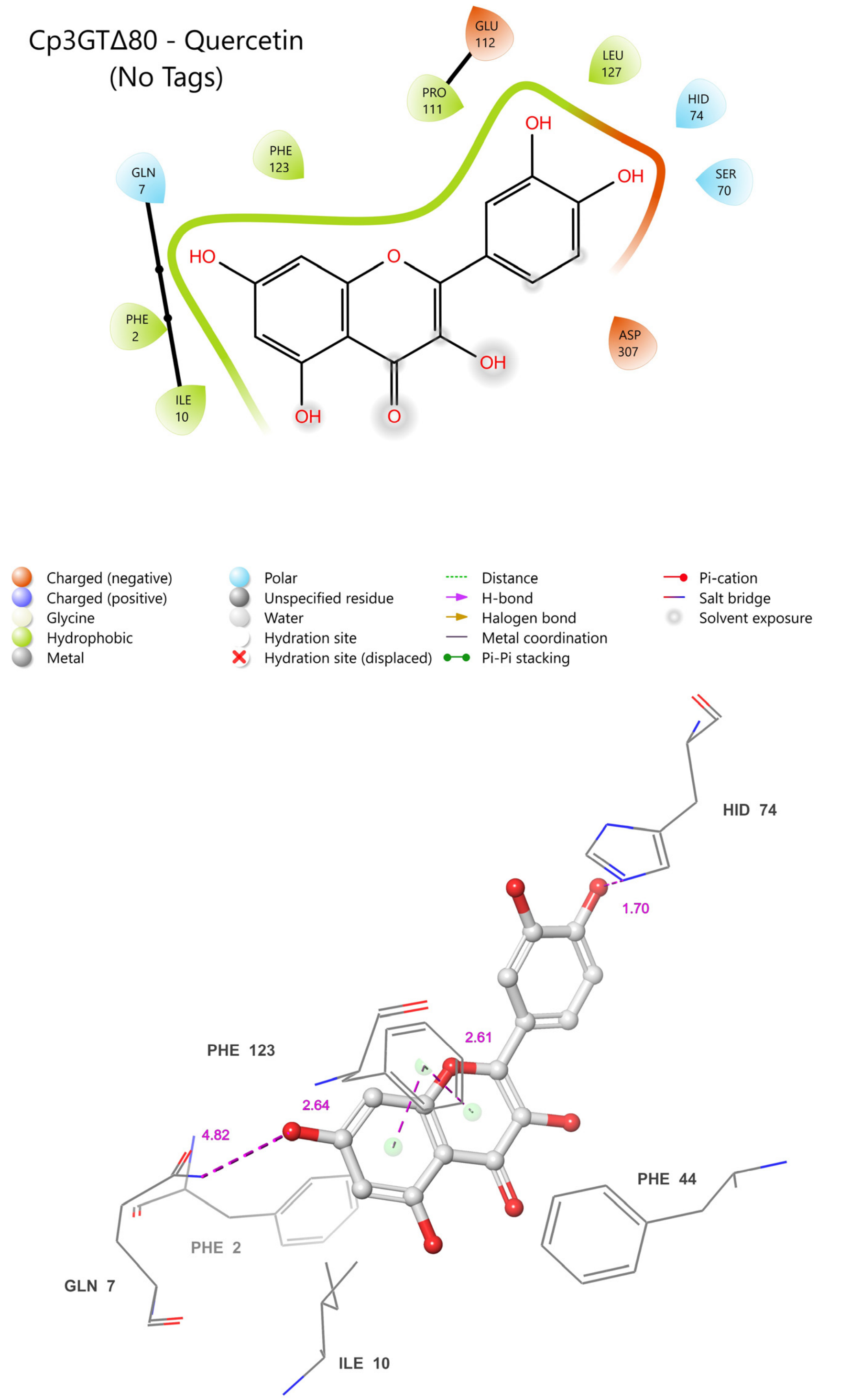

Figure S9: 2D And 3D Models of Cp3GTΔ80 (Modeled Without Tags) Residue-Substrate Interaction with Quercetin. All residues shown are positionally equivalent to Cp3GT shown in Figure S3 minus the 80 removed residues.

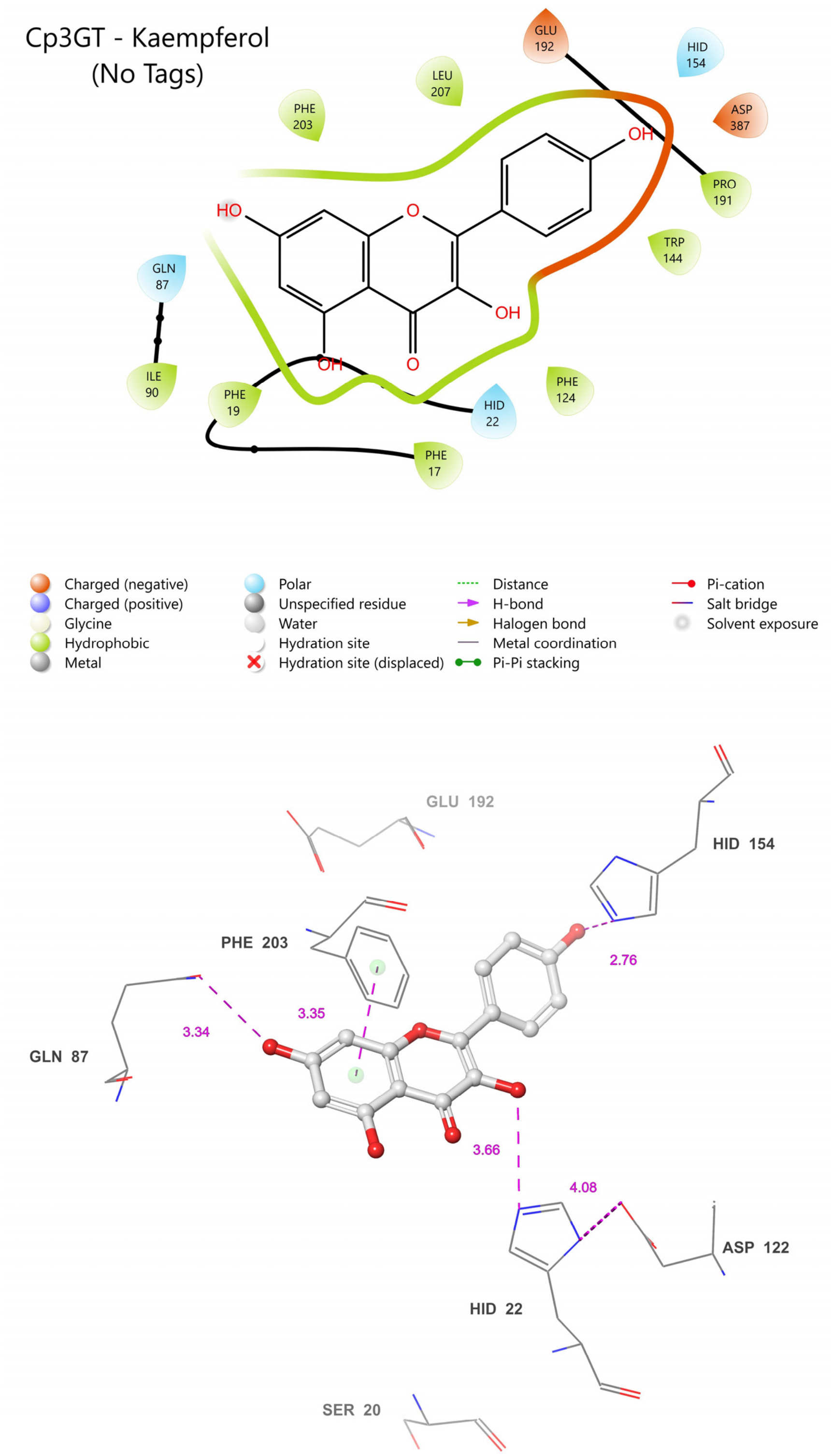

Figure S10: Visual Representation of Cp3GT (Modeled Without Tags) Residue-Substrate Interaction with Kaempferol. 2D (top) and 3D (bottom) schematic of Cp3GT bound with kaempferol derived from COFACTOR.

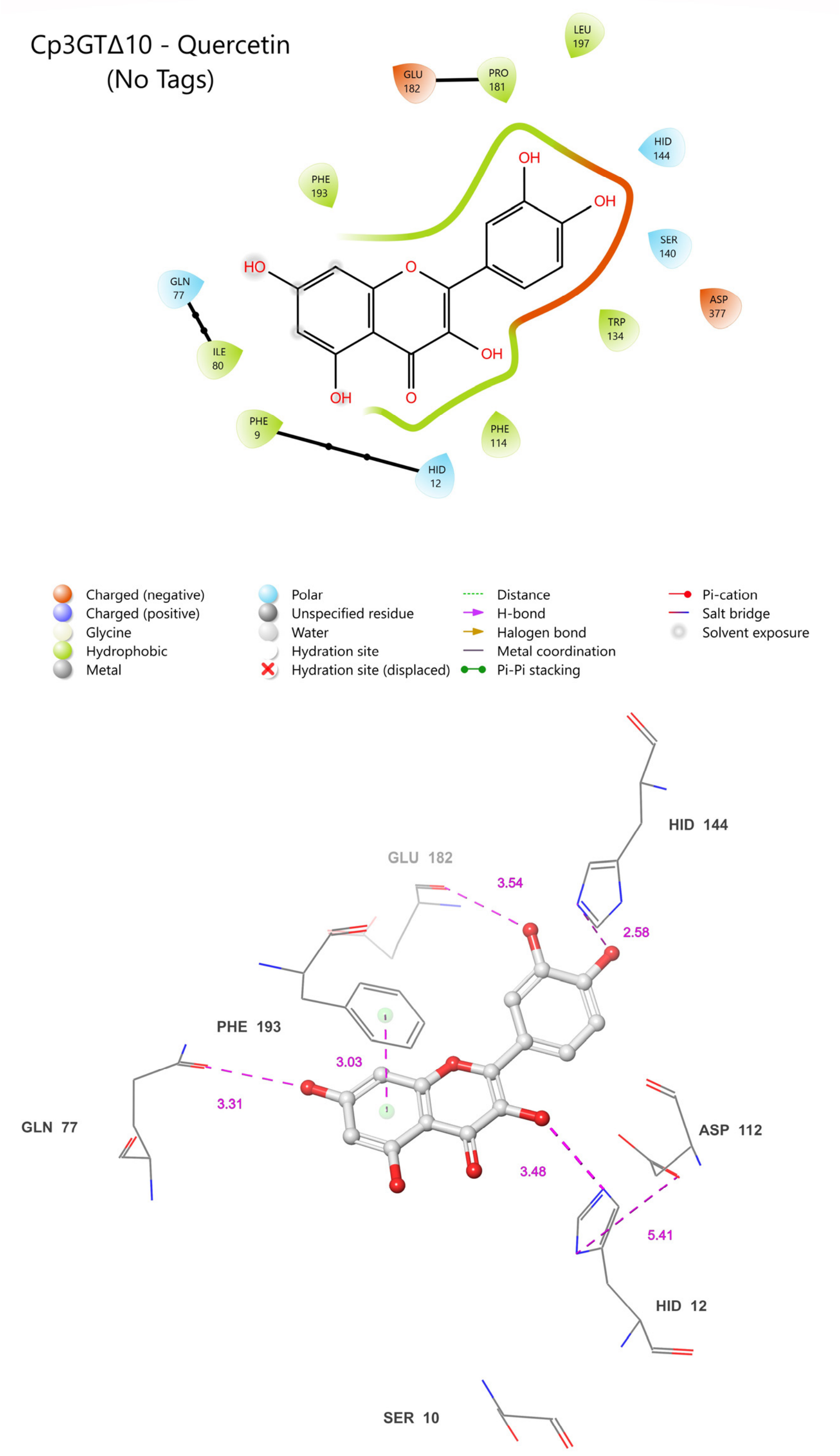

Figure S11: Visual Representation of Cp3GTΔ10 (Modeled Without Tags) Residue-Substrate Interaction with Quercetin. 2D (top) and 3D (bottom) schematic of Cp3GTΔ10 docked with quercetin derived from COFACTOR. All residues shown are positionally equivalent to Cp3GT shown in Figure S3 minus the 10 removed residues.
